# SIRT1–SIRT7 in Diabetic Kidney Disease: Biological Functions and Molecular Mechanisms

**DOI:** 10.3389/fendo.2022.801303

**Published:** 2022-05-13

**Authors:** Wenxiu Qi, Cheng Hu, Daqing Zhao, Xiangyan Li

**Affiliations:** ^1^Jilin Ginseng Academy, Key Laboratory of Active Substances and Biological Mechanisms of Ginseng Efficacy, Ministry of Education, Jilin Provincial Key Laboratory of Bio-Macromolecules of Chinese Medicine, Changchun University of Chinese Medicine, Changchun, China; ^2^College of Laboratory Medicine, Jilin Medical University, Jilin City, China

**Keywords:** biological function, signaling pathway, diabetes kidney disease, pathological process, sirtuins

## Abstract

Diabetic kidney disease (DKD) is a severe microvascular complication in patients with diabetes and is one of the main causes of renal failure. The current clinical treatment methods for DKD are not completely effective, and further exploration of the molecular mechanisms underlying the pathology of DKD is necessary to improve and promote the treatment strategy. Sirtuins are class III histone deacetylases, which play an important role in many biological functions, including DNA repair, apoptosis, cell cycle, oxidative stress, mitochondrial function, energy metabolism, lifespan, and aging. In the last decade, research on sirtuins and DKD has gained increasing attention, and it is important to summarize the relationship between DKD and sirtuins to increase the awareness of DKD and improve the cure rates. We have found that miRNAs, lncRNAs, compounds, or drugs that up-regulate the activity and expression of sirtuins play protective roles in renal function. Therefore, in this review, we summarize the biological functions, molecular targets, mechanisms, and signaling pathways of SIRT1–SIRT7 in DKD models. Existing research has shown that sirtuins have the potential as effective targets for the clinical treatment of DKD. This review aims to lay a solid foundation for clinical research and provide a theoretical basis to slow the development of DKD in patients.

## 1 Introduction

Diabetes mellitus (DM) is a metabolic disorder with chronic microvascular and macrovascular complications. DM is one of the most problematic health issues of the 21st century due to its severe complications. DM affects approximately 451 million people worldwide and is projected to reach 693 million by 2045 (1). NAD^+^ plays a key role in redox and energy metabolism. NAD^+^ acts as a co-substrate in the deacetylation reactions of sirtuins, and the regulation of the NAD^+^-sirtuins axis is a pivotal pathway for the new therapies of metabolic diseases (2). Moreover, in different renal disease models, such as diabetic kidney disease (DKD), sirtuins have been proven to regulate anti-fibrosis and anti-oxidative stress functions, and maintain the glomerular barrier integrity (3). DKD, diabetic retinopathy, and diabetic peripheral neuropathy are the main complications of DM, among which DKD has attracted worldwide attention due to its high incidence (20%–40% in diabetic patients) and poor prognosis (4, 5). DKD is a chronic disease that leads to renal failure; the treatments for rena0l failure are dialysis and kidney transplantation (6). However, once the disease progresses to end-stage renal disease, the course of this disease is both uncontrollable and irreversible ( (7). Although many researchers have studied the molecular mechanism of DKD and attempted to improve treatment strategies, DKD remains a clinically intractable complication of DM.

Histone deacetylases (HDACs) in eukaryotes are divided into IV classes, among which the I, II, and IV groups depend on Zn^2+^, whereas class III sirtuins depend on NAD^+^ to exert catalytic activity (8). The sirtuin family is classified into SIRT1–SIRT7 based on differences in the core structural domain, all of which catalyze the deacetylation of N^ℇ^-acyl-lysine on histone and non-histone substrates ( (9, 10). SIRT1, SIRT6, and SIRT7 are mainly found in the nucleus, SIRT2 is localized in the cytoplasm, and SIRT3, SIRT4, and SIRT5 are found in mitochondria, and their positions are not fixed (11). Sirtuins are involved in the regulation of various biological activities, including DNA repair, apoptosis, cell cycle, oxidative stress, metabolism, lifespan, and aging (12, 13). Based on biological regulatory functions, many studies have shown that the sirtuin family has therapeutic effects in many diseases. Sirtuins are pharmacological targets in neurodegenerative diseases, including Alzheimer’s disease, Parkinson’s disease, and Huntington’s disease (14). Moreover, the regulation of sirtuins reveals a complex network of cellular metabolism and will provide clues for the diagnosis, treatment, and prevention of cancer (15). Additionally, as mitochondrial sirtuins affect many aspects of mitochondrial metabolism and signal transduction, targeting sirtuins may represent a potential therapeutic target to combat age-related mitochondrial recession (16). Through reviewing the literature, we found many studies on sirtuins and DKD, but a lack of systematic and detailed summaries. Therefore, in this review, we have first introduced the biological regulatory functions of SIRT1–SIRT7 in DKD animal and cell models. Subsequently, we have summarized the signaling pathways for treating DKD with various treatments, and finally, examined the differences and clinical implications of sirtuins in DKD studies.

## 2 Search Strategy

Data for this review were identified by searching PubMed and Web of Science using the search terms “histone deacetylase”, “sirtuins”, “SIRT”, “diabetic nephropathy”, “diabetic kidney disease”, and “diabetic complication” for collecting articles from 2004 to 2021, with the language limited to English.

## 3 Pathological Process of DKD

The pathogenesis of DKD is multifactorial, involving structural, physiological, hemodynamic, and inflammatory processes, which ultimately lead to a decreased glomerular filtration rate (17). Hyperglycemia and hypertension are critical factors in the development of DKD (17). Proteinuria is an important factor in the development of DKD, which is directly and predictably associated with kidney damage (18). Proteinuria results from an abnormal permeability function of the glomerular filtration barrier, which consists of three layers of glomerular endothelial cells, the glomerular basement membrane (GBM), and podocytes (19). DKD is a microvascular complication of DM that develops from micro-proteinuria to massive proteinuria, ultimately leading to end-stage renal disease (18). Importantly, metabolic and hemodynamic changes in DM cause ultrastructural changes in the glomerular filtration barrier, including podocyte foot process fusion and separation, GBM thickening, reduction of endothelial cell glycocalyx, accumulation of mesangial extracellular matrix, and glomerular sclerosis, all of which are directly related to the increase in proteinuria (20).

### 3.1 Relationship Between the Expression of SIRT1–SIRT7 and DKD

The important role of SIRT1 has been demonstrated by the enhanced mitochondrial damage in *SIRT1* knockdown mice with DM, and its role in maintaining kidney cell homeostasis under mitochondrial stress or damage (21). Moreover, in advanced glycation end products (AGE)-treated rat primary glomerular mesangial cells (GMCs), investigators found that the overexpression of SIRT1 protected against reactive oxygen species (ROS) production and fibrosis by enhancing the Keap1/Nrf2/ARE pathway (22). Additionally, under the condition of HG-induced HK-2 cells, the deacetylase activity of SIRT1 decreased and resulted in renal tubular injury induced by the SIRT1/NF-κB/microR-29/Keap1 signaling pathway (23).

Furthermore, a reduction in the NAD^+^/NADH ratio has been shown to induce a decrease in SIRT3 activity and enhance mitochondrial oxidative stress in a DKD rat model (24). Another investigator found that the overexpression of SIRT3 antagonizes apoptosis in HG-induced HK-2 cells *via* the AKT/FOXO1 and AKT/FOXO3a signaling pathways (25). Similarly, in a streptozotocin (STZ)-induced mouse model, high expression of SIRT3 inhibited aberrant glycolysis and prevented fibrosis *via* the activation of PKM2 dimer formation and HIF-1α accumulation (26). Moreover, in HG-induced endothelial cells, the overexpression of SIRT3 activated the AMPK/SIRT3 pathway to sustain redox balance and alleviate vascular inflammation (27).

A previous report indicated that the overexpression of SIRT4 reduced the inflammatory effect and restrained apoptosis and the production of ROS in HG-induced mouse podocytes *via* the mitochondrial pathway (28).

In HG-induced podocytes, the overexpression of SIRT6 reduced mitochondrial dysfunction and apoptosis by activating the AMPK pathway (29). Another report illustrated that overexpression of SIRT6 promoted M2 macrophage transformation and alleviated kidney injury in *in vivo* and *in vitro* DKD models by upregulating the expression of Bcl−2 and CD206, and reducing the expression of Bax and CD86 (30). Additionally, another study demonstrated that in db/db mice and AGE/HG-induced human podocytes, overexpression of SIRT6 showed anti-apoptosis and anti-inflammatory effects by inhibiting the Notch pathway (31).

Taken together, these findings indicate that the overexpression of SIRT1, SIRT3, SIRT4, and SIRT6 reduces the biological impairment of kidney function in DKD models.

### 3.2 Gene Polymorphism and Clinical Research of Sirtuins in DKD

Human gene polymorphism plays an important role in elucidating the susceptibility and tolerance of the human body to diseases and poisons, the diversity of clinical manifestations of diseases, and the response to drug therapy (32–34). Studies have shown that SIRT1 and FOXO1 play important roles in the pathogenesis of DKD. Single nucleotide polymorphisms were analyzed by including 1066 patients with type 2 diabetes (T2DM) (413 without DKD and 653 with DKD), and the results indicated that the *SIRT1* gene variant rs10823108 and the *FoxO1* gene variant rs17446614 may be associated with DKD in patients with T2DM (35). Another study of gene polymorphisms suggested that, among 1016 patients with T2DM (388 without DKD and 628 with DKD), the transcriptional coactivator p300 rs20551 polymorphism is associated with the development of DKD, and the SIRT1 polymorphism is related to albumin-creatinine ratio progression (36). The researchers analyzed changes in serum vash-1 and other biomarkers in 692 patients with T2DM, and found that the UACR, VASH-1, HbA1c, ESR, CRP, VEGF, HIF-1α, TNF-α, and TGF-β1 levels in all patient groups were significantly higher, and the SIRT1 levels were lower compared to healthy controls. These findings indicated that serum VASH-1 may be associated with the expression of renal inflammation and fibrosis-related factors and have a potential connection with DKD (37). Another two-center, randomized study evaluated 117 patients with stage 2–4 DKD who were treated with sevelamer carbonate. The results showed that sevelamer carbonate increased anti-inflammatory defenses, including nuclear factor like-2, AGE receptor 1, and SIRT1, and decreased pro-inflammatory cytokines, such as TNF receptor 1 (38). In the latest clinical study, 313 patients with T2DM, 102 pre-diabetic patients, and 100 healthy volunteers were selected to study the relationship between SIRT6 and glucolipid metabolism and urinary protein. The clinical study results showed that SIRT6 increased with glucolipid metabolism and urinary protein markers, and is therefore expected to be a potential biomarker for the early prediction and diagnosis of glucolipid metabolism disorders and related nephropathy (39). The results of the above gene polymorphism and clinical studies indicate that sirtuins may represent a molecular target to explore new therapeutic approaches for DKD in the clinic.

## 4 Biological Effects of SIRT1–SIRT7 in DKD Models

**Figure 1:**
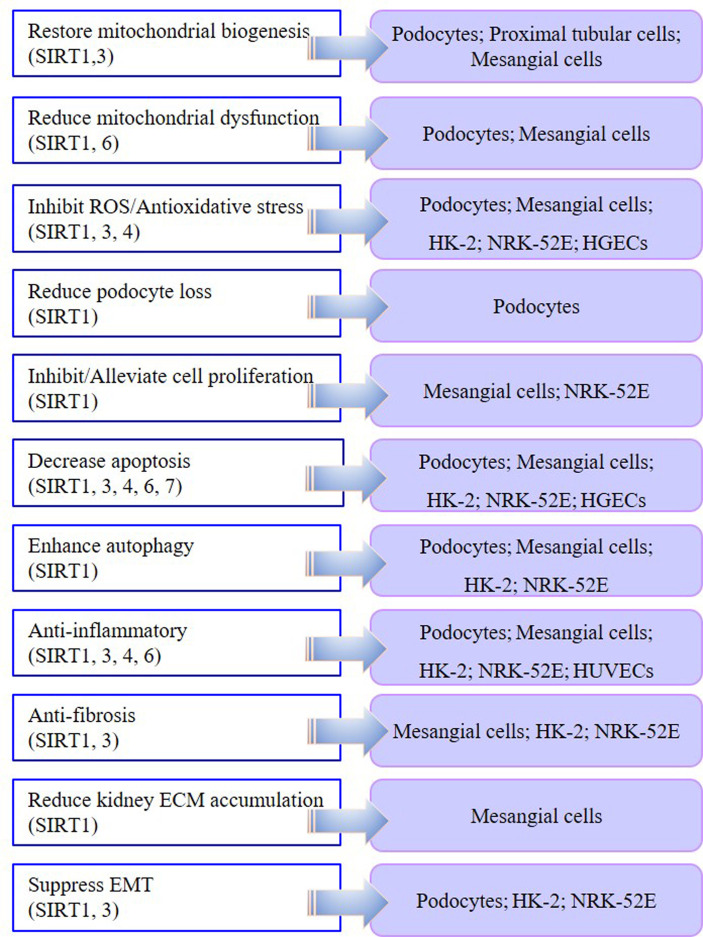
Biological role of SIRT1–SIRT7 in different DKD cell models.

In the cell models of DKD, injury models are mostly induced by HG or AGE, while most kidney fibrosis models are induced by TGF-β1 or HG in podocytes, mesangial cells, renal tubular cells, and some endothelial cells (**Table 1**). In podocyte, proximal tubular cell, and mesangial cell models, SIRT1 and SIRT3 are involved in the mechanism by which therapeutic drugs restore mitochondrial biosynthesis. In podocyte and mesangial cell models, SIRT1 and SIRT6 play significant roles in reducing abnormal mitochondrial function. Moreover, SIRT1, SIRT3, and SIRT4 are involved in the anti-oxidative stress effect in podocytes, mesangial cells, and renal tubular cells. SIRT1, SIRT3, SIRT4, SIRT6, and SIRT7 all participate in reducing the apoptosis of podocytes, mesangial cells, and renal tubular cells in DKD models. In most DKD cell models, therapies targeting SIRT1, SIRT3, SIRT4, and SIRT6 have shown anti-inflammatory effects. In DKD tubular cell models, both SIRT1- and SIRT3-targeted therapies displayed anti-fibrosis effects and suppressed epithelial-mesenchymal transition (EMT). Targeting SIRT1 also enhanced autophagy in various DKD models. By summarizing the results of previous research, we found that SIRT1, SIRT3, SIRT4, SIRT6, and SIRT7 play different biological functions in DKD cell models. Notably, SIRT1 is the most widely investigated HDAC with the most diverse biological functions **(** **Figure 1****)**.

**Table 1:** Cellular model of diabetic nephropathy used to study SIRT1-SIRT7.

Name		Species	Model
Podocytes		Human/Rat/Mouse	HG, AGE, ADR
**Mesangial cells**	GMCs (Glomerular mesangial cells)	Rat	HG, AGE
	HBZY-1	Rat	HG
	HMCs (Human mesangial cells)	Human	TGF-β, HG
	HRMCs (Human renal mesangial cells)	Human	HG
	Mouse mesangial cells	Mouse	HG
	mRMCs (Renal mesangial cells)	Mouse	HG
	NMS2	Rat	HG
	Raw264.7	Mouse	HG
	SV40 MES 13	Mouse	\
**Renal tubule**	BUMPT cells (Proximal tubule-derived cell line)	Mouse	HG
	HK-2 (Proximal tubule epithelial cell)	Human	TGF-β, HG
	mProx (Proximal tubular cells)	Murine	H_2_O_2_
	NRK-52E (Renal tubular epithelial cells)	Rat	HG, AGE
	RPTCs (Renal proximal tubule epithelial cells)	Human	HG
**Others**	HGECs (Human glomerular endothelial cells)	Human	HG
	HUVECs (Human umbilical vein endothelial cells)	Human	HG, AGE
	LLC-PK1 (Renal epithelial cell line)	Porcine	HG

Animal models are valuable for studying the pathological origins of human diseases because they allow in-depth investigation of mechanisms, which cannot be explored in clinical studies. As DKD animal models, db/db mice or rats, STZ and/or HFD-induced mice or SD/Wistar rats, and some unique transgenic mouse models are often used as research objects. We summarized the research methods of SIRT1, SIRT3, SIRT4, SIRT6, and SIRT7 in different DKD animal models to understand the methods of animal models more intuitively (**Tables 2****–** **9**).

**Table 2:** DKD studies on AMPK/sirtuins/PGC-1α pathway.

Reference	Drug/Target	Sirtuins	Model	Mechanism of protection	Pathway
(21)	SIRT1 Deficiency	SIRT1 Knockdown	SIRT1RNAi transgenic mouse STZ-induced mouse ADR-induced nephropathy murine podocytes	Enhance mitochondrial damage	SIRT1 signaling
(27)	SIRT3 OE	SIRT3	HG-induced HUVEC	Sustain redox balance and alleviate vascular inflammation	Increased SIRT3-activated AMPK pathway
(29)	SIRT6 OE	Up-regulate SIRT6	STZ induced male C57BL/6 mice HG-induced podocyte	Attenuate mitochondrial dysfunction and apoptosis	Activate AMPK pathway
(40)	Pro-renin receptor shRNA	Restore SIRT1	STZ C57BL/6 mouse HG-mouse renal mesangial cells (mRMCs)	Restore mitochondrial biogenesis and function	AMPK/SIRT1/PGC-1α signaling pathway
(41)	Resveratrol	Restore SIRT1 expression	db/db mice HG-induced NMS2 mesangial cells	Anti-apoptosis and oxidative stress	AMPK/SIRT1/PGC-1α axis
(42)	OP	Increase expression of SIRT1	HFD and STZ-induced mice	Suppress apoptosis and oxidative stress	Activate AMPK/SIRT1/PGC-1α signaling axis
(43)	CL316,243	Reverse the decrease of SIRT1	STZ and HFD treated mouse	Improve renal fibrosis, inflammation, and oxidative stress, and enhance BAT activity	AMPK/SIRT1/PGC-1α signaling pathway
(44)	Glycyrrhizic acid	Restore SIRT1	Male diabetic db/db mouse	Inhibit ROS	Activate AMPK/SIRT1/PGC-1 signaling
(45)	Resveratrol	Restore SIRT1 expression	db/db diabetic mouse HG-induced HGECs	Inhibit oxidative stress and apoptosis	By activating the AMPK/SIRT1/PGC-1α axis
(46)	GSPB2	Restore SIRT1 expression	HG−induced podocyte	Reduce mitochondrial dysfunction and apoptosis	Via the AMPK/SIRT1/PGC-1α axis
(47)	Grape seed procyanidin B2 (GSPB2)	Restore SIRT1 expression	High-dose glucosamine rat mesangial cells	Ameliorate mitochondrial dysfunction and inhibit apoptosis	The activation of the AMPK/SIRT1/PGC-1α axis
(48)	FGF21	Increase SIRT1 levels	OVE26 transgenic mouse as a T1DM nephropathy model	Anti-apoptosis, antioxidative stress, anti-inflammatory	AMPK/SIRT1 pathway
(49)	Metformin	Increase SIRT1 protein expression	HG-induced primary rat podocytes	Improve the insulin resistance	Dependent on AMPK and SIRT1 activity
(50)	Salidroside	Restore SIRT1 expression	STZ-induced Wistar male rat as T1DM model HG-induced rat mesangial cells	Anti-apoptosis and oxidative stress	Activate AMPK/SIRT1 signaling pathway
(51)	Roflumilast	Restore SIRT1 expression	STZ-induced SD rat	Anti-apoptosis	AMPK/SIRT1 pathway
(52)	Catalpol	Increase SIRT1 level	HFD/STZ-induced mice, HG-induced podocyte model	Inhibit oxidative stress and inflammation accompanied with pyroptosis	Activate AMPK/SIRT1/NF-κB pathway
(53)	Geniposide (GE)	Up-regulate protein expression of SIRT1	HFD/STZ-induced mice HG-induced podocyte model	Antioxidative stress, anti-inflammatory	APMK/SIRT1/NF-κB pathway
(54)	Stanniocalcin-1	Restore SIRT3 protein expression	Male C57BL/6J db/db mice, HG-treated BUMPT cells	Antioxidant and anti-apoptotic activities	AMPK/SIRT3 pathway
(55)	Metformin	Restore SIRT1	HFD and low dose STZ rats HG-induced RMCs	Alleviate oxidative stress and enhance autophagy	AMPK/SIRT1/FOXO1 pathway
(56)	Cocoa	Restore SIRT1	Zucker diabetic fatty (ZDF) rats	Antioxidant, stimulate autophagy and suppress apoptosis	Activation of stress related key proteins (ERK/MAPKs and NOX-4), cytoprotective-related proteins (AMPK, SIRT1 and mTOR), autophagy and apoptosis pathways
(57)	Probucol	Restore SIRT1 expression	STZ-induced mouse HG-induced HK-2	Attenuate oxidative stress and fibrosis	Suppress p66Shc expression via the AMPK/SIRT1/AcH3 pathway
(58)	Glycyrrhizic acid	Restore SIRT1 expression	HG-induced NRK-52E	Anti-cell proliferation and oxidative stress	Increase AMPK, SIRT1 and Mn-SOD expression
(59)	Resveratrol	No change on SIRT1 expression	Male db/db mouse H_2_O_2_ exposed murine proximal tubular cells (mProx)	Improve oxidative stress and enhance mitochondrial biogenesis	Via AMPK/SIRT1-independent pathway
(60)	Theobromine	SIRT1 activation	STZ-induced male spontaneously hypertensive rats HG-induced iHMCs	Reduce kidney ECM accumulation	AMPK activation
(61)	Honokiol	Activation of SIRT3	BTBR ob/ob mice with T2DM	Preserve mitochondrial wellness	Through the activation of SOD2 and the restoration of PGC-1α expression
(62)	Resveratrol	Restore SIRT1 expression	STZ induced CD-1 mouse HG-induced mouse podocytes	Attenuate mitochondrial oxidative stress	Via SIRT1/PGC‐1α pathway
(63)	Salidroside	Restore SIRT1 expression	STZ induced male C57BL/6J mouse	Inhibit fibrosis	SIRT1/PGC-1α axis
(64)	BF175 (SIRT1 agonist)	Increase SIRT1 activity	OVE26 mouse HG induced human podocytes	Reduce podocyte loss and oxidative stress	Deacetylation of PGC-1α and activation of PPARγ
(65)	Formononetin	Increase SIRT1 expression	HFD and low dose of STZ induced rat	Anti-oxidative stress	Increase SIRT1 expression
(66)	Glucagon−like peptide−1	Restore SIRT1	HG-induced mouse podocytes	Reduce apoptosis, ROS, and proinflammatory cytokine	Activation of SIRT1
(67)	Resveratrol	Enhance SIRT1 expression	STZ-induced T2DM rat HG-induced NRK-52E cells	Suppress apoptosis through promoting autophagy activity	SIRT1 activation
(68)	lncRNA GAS5 OE	Enhance the expression level of SIRT1	HG induced mesangial cells (RAW264.7)	Inhibit cell proliferation and fibrosis	By sponging miR-221 and modulating SIRT1 expression
(69)	LncRNA SOX2OT OE	Increase SIRT1 expression	HG-induced human podocytes cells (HPCs)	Induce autophagy	miR-9/SIRT1 axis
(70)	Downregulation of miR-138	Bind the 3'-UTR of SIRT1	HG-induced mice podocytes db/db mice kidney tissues	Anti-inflammatory	The regulatory axis of miR-138/SIRT1/p38/TTP
(142)	Olmesartan	Restore SIRT1 expression	db/db mouse HG-induced podocytes	Inhibit podocyte apoptosis	Through inhibiting angiotensin II/p38/SIRT1
(143)	Selenium nanoparticles	Up-regulate SIRT1	STZ induced SD male rat	Anti-oxidative stress and lower apoptosis	Activate HSP-70/SIRT1 axis
(144)	hnRNP F OE	Increase SIRT1 expression	db/db hnRNP F-Tg mouse HG-induced rat IRPTCs	Against oxidative stress, tubulointerstitial fibrosis, and RPTC apoptosis	Via stimulation of SIRT1 expression and signaling
(145)	KD miR-133b and miR-199b	Upregulate SIRT1	Old male OLETF rat (spontaneous T2DM) TGF-β1-treated HK-2	Attenuate EMT and renal fibrosis	By targeting SIRT1
(146)	Inhibiting PARP1	Upregulate the expression of SIRT1	db/db mice HG-induces mesangial cells	Decrease kidney ECM accumulation	AMPK/PGC-1α signaling pathway

Generally, these abnormal manifestations, such as inflammation, oxidative stress, abnormal mitochondrial function, renal fibrosis, podocyte loss and apoptosis, and impaired autophagy, are all likely to occur during the development of DKD. Meanwhile, SIRT1, SIRT3, SIRT4, SIRT6, and SIRT7 play diverse regulatory roles in these physiological processes.

## 5 The Role of SIRT1–SIRT7 in Signaling Pathways in DKD Models

### 5.1 AMPK/Sirtuins/PGC-1α Pathway

AMPK and SIRT1 are the two main energy sensors, which directly affect the activity of PGC-1α through phosphorylation and deacetylation, respectively (40). Studies have shown that impaired renal function under HG is directly related to the inactivation of the AMPK/SIRT1/PGC-1α signaling pathway (41). The study results showed that CL316, 243, glycyrrhizic acid, and a polysaccharide from okra (OP) all played antioxidant roles, reduced inflammation, and improved fibrosis through activation of the AMPK/SIRT1/PGC-1α pathway in STZ and/or HFD-induced db/db DKD mouse models (42–44). Resveratrol, pro-renin receptor shRNA, and grape seed procyanidin B2 (GSPB2) regained SIRT1 expression *via* the AMPK/SIRT1/PGC-1α signaling axis in DKD models, thus restoring mitochondrial biosynthesis and function, reducing oxidative stress, and inhibiting apoptosis (40, 41, 45–47). In DKD animal or cell models, FGF21, metformin, salidroside, and roflumilast increased or restored the expression level of SIRT1 and played anti-apoptotic and anti-oxidative roles by activating the AMPK/SIRT1 pathway (48–51). Moreover, catalpol and geniposide (GE) up-regulated the expression of SIRT1 in DKD models and inhibited oxidative stress and inflammation by activating the AMPK/SIRT1/NF-κB pathway (52, 53). Additionally, in HG-induced renal tubule cells, restoration of SIRT3 expression through stanniocalcin-1 activated the AMPK/SIRT3 pathway to produce antioxidant and anti-apoptotic activities (54). Furthermore, cocoa, metformin, glycyrrhizic acid, and probucol restored SIRT1 expression by activation of the AMPK/SIRT1 pathway, ultimately reducing oxidative stress, apoptosis, and enhancing autophagy in DKD models (26, 55–58). However, one particular study reported that resveratrol improved oxidative stress and enhanced mitochondrial biogenesis without altering SIRT1 expression, and is independent of the AMPK/SIRT1 pathway. The distinction is that they used H_2_O_2_-exposed proximal tubular cells as a DKD model, as opposed to HG or AGE, which are more commonly used (59). In HG-induced immortalized human mesangial cells (iHMCs), theobromine could activate SIRT1 and decrease kidney extracellular matrix (ECM) accumulation by activating the AMPK pathway (60). In BTBR ob/ob mice, honokiol protected mitochondrial health by activating mitochondrial SIRT3, which first revealed the renal protective effect of SIRT3 on diabetic glomerular disease (61). Moreover, in STZ-induced mouse models, salidroside and resveratrol restored SIRT1 expression *via* the SIRT1/PGC‐1α pathway, thus inhibiting fibrosis and reducing mitochondrial oxidative stress, respectively (61–63). BF175, as an activator of SIRT1, increased SIRT1 activity to acetylate PGC‐1α and activate PPARγ to reduce podocyte loss and oxidative stress (64). Furthermore, glucagon−like peptide−1, formononetin, and resveratrol enhanced SIRT1 expression in DKD models to attenuate apoptosis and oxidative stress by activating SIRT1 (65–67). Beyond this, in HG-induced podocytes or mesangial cells, overexpression of lncRNA SOX2OT, overexpression of lncRNA GAS5, or downregulation of miR-138 increased SIRT1 expression or activity to induce autophagy, inhibit fibrosis, and decrease inflammation, respectively, by regulating the miR-9/SIRT1, miR-221/SIRT1, and miR-138/SIRT1 axes (68–70). Through the studies reported above, we conclude that AMPK/Sirtuins/PGC-1α is a crucial pathway in regulating the pathological process of DKD (**Table 2**).

### 5.2 SIRT1/p53 Pathway

SIRT1 specifically associates with and acetylates the tumor suppressor protein p53, thereby negatively regulating p53-mediated transcriptional activation. More importantly, p53 deacetylation by SIRT1 prevents DNA damage and stress-induced cell senescence and apoptosis (71, 72). A previous study has shown that in HG-induced podocytes or HK-2 cells, inhibition of miR-150-5p or miR-155-5p, which could bind to the 3’-UTR of SIRT1, promoted autophagy by targeting the SIRT1/p53 pathway (73, 74). Moreover, in DKD animal and cell models, H_2_S, resveratrol, and calcium dobesilate restored or enhanced SIRT1 expression to prevent apoptosis by activating the SIRT1/p53 pathway (75–77). These reports suggest that the SIRT1/p53 pathway reduces cellular stress in HG-induced cells or STZ-induced animals’ models (**Table 3**).

**Table 3:** DKD studies on SITR1/p53 pathway.

Reference	Drug/Target	Sirtuins	Model	Mechanism of protection	Pathway
(73)	Inhibition miR-155-5p	Binding to the SIRT1 3'UTR region	HG-induced HK-2	Promote autophagy	A signaling loop p53/miR-155-5p/SIRT1
(74)	Silencing of miR-150-5p	Targeted the 3'-UTR of SIRT1	HG-induced podocyte injury STZ-induced diabetic nephropathy in mice	Activate AMPK-dependent autophagy	Targeting SIRT1/p53/AMPK Pathway
(75)	H_2_S	Upregulate SIRT1	STZ induced male rat	Suppress oxidative stress and apoptosis	SIRT1, SOD, caspase-3, p53, MDA
(76)	Resveratrol	Restore SIRT1 expression	STZ-induced Wistar rat HG-induced HK-2	Inhibit apoptosis	SIRT1/p53 axis
(77)	Calcium dobesilate	Enhance SIRT1 signaling	Renal interstitial fibrosis induced by unilateral ureteral obstruction (UUO) mouse model HUVECs	Suppress EMT progression and promote anti-apoptotic	Via activating the SIRT1/p53 signaling pathway

### 5.3 SIRT1/NF-κB-Related Pathway

Previous studies have demonstrated that the ability of SIRT1 deacetylation is critical to control the function of the transcription factor NF-κB, as SIRT1 modulates various biological responses by deacetylating NF-κB, including inflammation and autophagy (78, 79). In DKD models, isoliquiritigenin (ISLQ), baicalin, astragaloside IV, ligustilide, nicotinamide mononucleotide (NMN), and Tangshen formula have been shown to increase or activate SIRT1 through the SIRT1/NF-κB signaling pathway to improve inflammation, decrease apoptosis, and enhance autophagy (80–86). Moreover, in STZ-induced mouse models, BF175 decreased albuminuria and glomerular disease *via* the transcription factor NF-κB and p53 pathways (87). Additionally, panax notoginseng saponins (PNS) and baicalin have been found to up-regulate SIRT1 to inhibit inflammation, reactivate autophagy, and alleviate fibrosis *via* the NF-κB and TGF-β pathways in DKD models (88, 89). Furthermore, in HG-induced HK-2 cells, Na_2_S_4_ has been shown to directly sulfhydrate two conserved domains of SIRT1, leading to dephosphorylation and deacetylation of NF-κB and STAT3, which improves oxidative stress, apoptosis, and the inflammatory response (90). Thus, SIRT1 also has a protective effect on renal function by regulating downstream of NF-κB in DKD (**Table 4**).

**Table 4:** DKD studies on SIRT1/NF-κB related pathway.

Reference	Drug/Target	Sirtuins		Model	Mechanism of protection	Pathway
(23)	----	SIRT1		STZ-induced Wistar rat HG-induced HK-2	Inhibit renal tubular injury	Via SIRT1/NF-κB/microR-29/Keap1 signal pathway
(80)	Baicalin	Increase the expression of SIRT1		HG-induced podocyte	Decrease apoptosis of high glucose induced podocyte	SIRT1/NF-κB signaling pathway
(81)	ISLQ	Restore SIRT1		Male rat by STZ	Antioxidant, anti-inflammatory, and reduce collagen accumulation	Normalize the SIRT1/NF-κB balance, control NLRP3 expression
(82)	Astragaloside IV	Increase SIRT1 expression		Polygenic KK-Ay mice models HG induced podocyte	Inhibit EMT and enhance autophagy	SIRT1/NF-κB pathway
(83)	Tangshen formula	Activate SIRT1		STZ+HFD induced SD rat	Improve inflammation	Through SIRT1/NF-κB pathway
(84)	NMN	Restore SIRT1 expression		STZ induced SD male rat HG induced HBZY-1	Alleviate inflammatory−fibrosis	Nampt/NF-κB p65 and SIRT1 signaling pathway
(85)	Astragaloside IV	Restore SIRT1 expression		HFD-induced KK-Ay mouse Mesangial cell (SV40 MES 13)	Enhance autophagy	SIRT1/NF-κB pathway
(86)	Ligustilide	Promote SIRT1 protein expression		STZ combined with a HFD rat	Attenuate podocyte injury	Suppressing the SIRT1/NF-κB signaling pathways
(87)	BF175	Increase SIRT1		STZ mice	Reduce albuminuria and glomerular disease	NF-κB and p53 signaling pathways
(88)	Baicalin	Enhance level of SIRT1		STZ rats	Inhibit inflammation, inhibit extracellular matrix accumulation, regulate cell proliferation, reactivate autophagy, alleviate renal fibrosis	NF-κB signaling pathway, TGF-β/Smad3 pathway, IGF-1/IGF-1R/p38 MAPK pathway
(89)	PNS	Up-regulate SIRT1	Alloxan-induced SD rat HG-induced RMCs		Inhibit inflammation and antioxidant	Through decreasing the NF-κB-mediated induction of inflammatory cytokines and TGF-β1
(90)	Na_2_S_4_	Sulfhydrating SIRT1	HG-induced HK-2 cells STZ mice		Restrain the overproduction of inflammation cytokine and ROS	Suppressing phosphorylation and acetylation of p65 NF-κB and STAT3

### 5.4 Sirtuins and the TGF-β1/Smad3 Pathway

TGF-β superfamily members are critical in regulating fibrosis in most chronic kidney diseases, and the inhibition of TGF-β1 or its downstream signaling (e.g. Smad) has been shown to decrease renal fibrosis (91–94). It has also been reported that the reduction of miR-34a-5p targets the 3’UTR of SIRT1, which inhibits fibrosis by regulating TGF-β1 signaling in HG-induced HK-2 cells (95). Moreover, in AGE stimulated NRK-52E cells, oligo-fucoidan has been shown to improve renal fibrosis *via* restraint of the pro-fibrosis process caused by TGF-β1 activation (96). Additionally, tetrahydroxystilbene glucoside (TSG) restored SIRT1 expression to alleviate oxidative stress by targeting SIRT1 and TGF-β1 signaling both *in vivo* and *in vitro* (97). Moreover, the inhibition of miRNA−135a−5p increased SIRT1 expression and inhibited fibrosis by targeting the TGF-β1/Smad3 pathway in TGF-β1-induced HK-2 and HMC cells (98). As a unique example, FOXO3a binds to the SIRT6 promoter and promoted SIRT6 expression to reduce EMT and fibrosis through FOXO3a-mediated SIRT6/Smad3 pathway in DKD models (99). The above summary highlights the vital function of the TGF-β1/Smad3 pathway in the regulation of renal fibrosis by sirtuins in DKD (**Table 5**).

**Table 5:** DKD studied on sirtuins and TGF-β1/Smad3 pathway.

Reference	Drug/Target	Sirtuins	Model	Mechanism of protection	Pathway
(95)	Reduce miR-34a-5p	Targeting the 3'UTR of SIRT1	HFD/STZ induced C57BL/6 mouse HG induced HK-2	Inhibit fibrosis	TGF-β1 signaling
(96)	Oligo-Fucoidan	Restore SIRT1 expression	AGE stimulated NRK-52E cells STZ and nicotinamide combined with a HFD mouse	Improve kidney disease caused by excessive fibrosis	Suppress the HMGB1/RAGE/NF-κB/TGF-β1/TGF-β1R/FN pathway and HIF-1α activation
(97)	TSG	Restore SIRT1 expression	STZ-induced SD rat HG-induced HBZY-1	Alleviate oxidative stress	SIRT1 and TGF-β1 pathway
(98)	Inhibition of miRNA−135a−5p	Target SIRT1 3'UTR	TGF-β-induced HK-2 and HMCs	Inhibit renal fibrosis	Target SIRT1 and inactivating Smad3 signaling
(99)	FOXO3a	Bind to the SIRT6 promoter and promote SIRT6 expression	db/db T2DM mouse HG-induced HK-2	Reduce EMT and fibrosis	FOXO3a-mediated SIRT6/Smad3 signaling pathways

### 5.5 PI3K/AKT/FOXO Pathway

The PI3K/AKT pathway plays a crucial role in cell physiology, which participates in glucose homeostasis, lipid metabolism, protein synthesis, and cell proliferation and survival (100, 101). FOXO1 and FOXO3a, as important substrates of AKT, are regulated by the PI3K/AKT pathway (102). Researchers have found that resveratrol restored SIRT1 expression to attenuate oxidative stress damage in STZ-induced rat models through the SIRT1/FOXO3a or SIRT1/FOXO1 pathway (103–105). Furthermore, fucoxanthin and angiotensin 1–7 restored SIRT1 expression in response to antioxidative stress *via* the AKT/SIRT1/FOXO3α and SIRT1/FOXO1/ATGL signaling pathways in DKD models, separately (106, 107). Moreover, in STZ- and HFD-induced mouse models, purinergic receptor (P2Y2R) deficiency enhanced autophagy and the expression of SIRT1 by AKT/FOXO3a and SIRT1 signaling pathways (108). Additionally, pyrroloquinoline quinine increased the expression of SIRT3 to antagonize oxidative stress and apoptosis in HG-induced HK-2 cells *via* the PI3K/AKT/FOXO3a signaling pathway (109). Moreover, it has been reported that progranulin (PGRN) restored both SIRT1 and SIRT3 to maintain mitochondrial biogenesis and mitophagy *via* SIRT1/PGC-1α/FOXO1 signaling in HG-treated podocytes (110). These findings suggest that the PI3K/AKT/FOXO pathway performs important biological functions in improving DKD by targeting sirtuins (**Table 6**).

**Table 6:** DKD studied on PI3K/AKT/FOXO3 pathway.

Reference	Drug/Target	Sirtuins	Model	Mechanism of protection	Pathway
(25)	SIRT3 OE	SIRT3	HG-induced HK-2	Antagonize high glucose-induced apoptosis	AKT/FoxO signaling pathway
(103)	Resveratrol and rosuvastatin	Restore SIRT1 mRNA expression	STZ-induced Wistar rat	Attenuate oxidative stress damage	Through increasing FOXO1/SIRT1 dependent antioxidant defenses
(104)	Resveratrol	Restore SIRT1 expression	STZ-induced Wistar rat HG-induced HK-2	Reduce oxidative stress damage	SIRT1/FOXO3a pathway
(105)	Resveratrol	Restore SIRT1 expression	STZ-induced SD rat	Anti-oxidative stress	SIRT1/FOXO1 pathway
(106)	Fucoxanthin	Restore SIRT1	GMCs cultured in HG	Antioxidative stress and anti-fibrosis	AKT/SIRT1/FOXO3a signaling
(107)	Angiotensin 1–7	Increase SIRT1 expression	db/db mouse T2DM model	Reduce oxidative stress, inflammation, and lipotoxicity	SIRT1/FOXO1/ATGL pathway
(108)	P2Y2R deficiency	Increased SIRT1 expression	HFD and STZ mouse	Enhance autophagy response	AKT/FOXO3a and SIRT1 signaling pathways
(109)	Pyrroloquinoline quinine	Upregulate SIRT3 expression	HG-induced HK-2	Anti-oxidative stress and apoptosis	PI3K/AKT/FOXO3a pathway
(110)	PGRN	Restore SIRT1 and SIRT3	STZ-induced mice and patients with DKD, HG-treated podocytes	Maintain mitochondrial biogenesis and mitophagy	Via PGRN/SIRT1/PGC-1α/FOXO1 signaling
(147)	Reduce LncRNA MALAT1	Restore SIRT1 expression	HG induced HK-2	Renal protective effect	MALAT1/FOXO1/SIRT1 signaling

### 5.6 Keap1/Nrf2/ARE Pathway

Dysregulation of Nrf2 transcriptional activity has been described in the pathogenesis of various diseases, and the Nrf2/Keap1 axis is a key regulator of cell homeostasis (111). It has been reported that formononetin, resveratrol, and polydatin up-regulate the expression of SIRT1 to anti-oxidative stress and fibrosis by activating the Nrf2/ARE pathway in HG/AGE-induced GMCs (112–114). Investigators have also found that SRT2104 (SIRT1 activators) protect against oxidative stress, inflammation, and fibrosis *via* the SIRT1/p53/Nrf2 pathway in DKD models (115). Moreover, in HG-induced NRK-52E cells, ISLQ treatment reduced inflammation and oxidative stress by inhibiting MAPK activation and the induction of Nrf2 signaling (116). These findings demonstrate that SIRT1 regulates the transcription factor Nrf2 in DKD models (**Table 7**).

**Table 7:** DKD studied on Keap1/Nrf2/ARE pathway.

Reference	Drug/Target	Sirtuins	Model	Mechanism of protection	Pathway
(22)	SIRT1 OE	SIRT1	AGEs-treated rat primary GMCs	Inhibit ROS production and anti-fibrosis	Enhanced the activity of Keap1/Nrf2/ARE pathway
(112)	Polydatin	Reverse the downregulation of SIRT1 protein expression and deacetylase activity	AGEs-induced GMCs	Anti-oxidative stress and fibrosis	Activation of SIRT1/Nrf2/ARE pathway
(113)	Formononetin	Up-regulated the expression of SIRT1	GMCs exposed to HG	Antioxidative stress, prevent the progression of renal fibrosis	Nrf2/ARE signaling pathway
(114)	Resveratrol (SIRT1 activator)	Restore SIRT1 expression	STZ-induced SD rat AGEs-induced SD rat primary GMCs	Antioxidative and fibrosis	By activating the Nrf2/ARE pathway
(115)	SRT2104	Enhance SIRT1 expression and activity	STZ induced C57BL/6 mouse	Protection against the oxidative stress, inflammation, fibrosis	SIRT1/p53/Nrf2 pathway
(116)	ISLQ	SIRT1 binds to ISL directly	STZ-induced T1DM HG-induced NRK-52E cells	Reduce inflammation and oxidative stress	Inhibition of MAPK activation, and the induction of Nrf2 signaling

### 5.7 STAT and HIF-1α-Related Pathway

It has been reported that connexin 43, LincRNA 1700020I14Rik, and silencing of miR-217 restrain inflammation and fibrosis in both *in vivo* and *in vitro* DKD models through SIRT1/HIF-1α signaling (117–119). Additionally, in AGE-induced human podocytes, PYR as an AGE inhibitor, restored SIRT1 expression to reduce kidney injury by decreasing p65 and STAT3 acetylation (120). In one study in HFD-diet DM rats, EX-527, as a SIRT1 inhibitor, reduced SIRT1 expression and increased SIRT3 expression to lessen fibrosis and inflammation by blocking the phosphorylation of EGFR and PDGFR, blocking STAT3 signaling (121). In another study, glucagon-like peptide-1 decreased SIRT1 expression to improve the inflammatory changes in db/db mice by inhibiting JAK/STAT signaling (122). Thus, STAT and HIF-1α-related pathways reduce negative effects in DKD models by targeting sirtuins (**Table 8**).

**Table 8:** DKD studied on STAT and HIF-1α pathway.

Reference	Drug/Target	Sirtuins	Model	Mechanism of protection	Pathway
(26)	SIRT3 high expression	SIRT3	STZ-induced mice model (fibrotic model: CD-1, less fibrotic model: C57Bl6	Inhibit aberrant glycolysis and combat fibrosis	By activation of PKM2 dimer formation and HIF-1α accumulation
(117)	Silencing of miR-217	Restore SIRT1 expression	HG-induced RMCs	Restrain inflammation and fibrosis	Through SIRT1/HIF-1α signaling pathway
(118)	LincRNA 1700020I14Rik	SIRT1	C57BL/KsJ db/db mouse HG induced mouse mesangial cells	Alleviate cell proliferation and fibrosis	miR-34a-5p/SIRT1/HIF-1α signaling
(119)	Connexin 43	Increase SIRT1 levels	db/db mice HG-induced NRK-52E cells	Inhibit the EMT progress and renal tubulointerstitial fibrosis	SIRT1/HIF-1α signaling pathway
(120)	PYR	Restore SIRT1 expression	db/db mouse AGE-induced human podocytes	Reduce kidney injury	Reduced p65 and STAT3 acetylation
(121)	EX-527	Reduce SIRT1 expression, increase SIRT3 expression	HFD-induced diabetic rats	Anti-fibrosis and anti-inflammation	Block the phosphorylation level of EGFR and PDGFR, blockade of STAT3 signaling
(122)	Glucagon-like peptide-1	Decrease SIRT1 expression	db/db mouse AGEs and HG induced HUVECs	Improve the inflammatory changes	Inhibit the JAK/STAT pathway

### 5.8 Other Pathways Involved in the Regulation of Sirtuins in DKD

#### 5.8.1 Pathways Associated With SIRT1 in DKD

Researchers have shown that both 1α, 25-Dihydroxyvitamin D3 and puerarin activate and increase SIRT1 expression to achieve anti-oxidative effects by suppressing NOX4 expression in DKD models (123, 124). Carnosine upregulated SIRT1 expression to decrease glycative and lipoperoxidative stress in HG-induced podocytes *via* the Hsp70/HO-1 pathway. Another report showed that anserine revealed anti-oxidant and glycative stress in HG-induced HK-2 cells *via* the Hsp70/HO-1 defense system, but did not affect SIRT1 expression (125, 126). Several other studies have shown that aerobic exercise training, inhibition of HIC1, INT-767 (FXR/TGR5 dual agonist), and SGLT2 restored SIRT1 expression under DKD animal and cell models, which improve mitochondrial function, reduce ROS, anti-inflammation, and prevent glucose entry (127–130). These results suggest that SIRT1 largely exhibits anti-inflammatory and anti-oxidant effects through different signaling pathways in DKD models.

#### 5.8.2 Pathways Associated With SIRT3 in DKD

Apigenin (CD38 inhibitor) and empagliflozin (SGLT2 inhibitor) have been shown to increase SIRT3 levels in HG-induced HK-2 cells to relieve mitochondrial oxidative stress and restore aberrant functions; this is mediated by restoring the NAD^+^/NADH ratio and inhibiting glucose uptake into the proximal tubules, respectively (131, 132). Liraglutide (glucagon−like peptide−1 agonist) has also been shown to increase SIRT3 expression to prevent the activation of mitochondrial apoptosis by activating the ERK−Yap signaling pathway in HG-induced HRMCs (133). It has been reported that INT-777 (TGR5-agonist) increased the activity of both SIRT1 and SIRT3 to improve mitochondrial biogenesis, and reduce oxidative stress and fibrosis *via* the TGR5 pathway in db/db diabetic mice (134). Moreover, in the C57BL/KsJ db/db mouse model, the overexpression of SIRT3 reduced apoptosis and fibrosis through modulation of mitophagy (135). It can be seen from the above results that high expression of SIRT3 reduced mitochondrial stress response, including oxidative stress and apoptosis.

#### 5.8.3 Pathways Associated With SIRT6 in DKD

SIRT6-knockout male mice have been shown to exhibit an enhanced fibrotic phenotype, which was controlled by the Nampt-SIRT6 axis to regulate extracellular matrix remodeling, and the authors found that SIRT1 is not the controller of SIRT6 expression (136). The results of this article show that SIRT6 plays an important regulatory role in ECM remodeling.

#### 5.8.4 Pathways Associated With SIRT7 in DKD

In HG-treated podocytes, the increase in SIRT7 has been shown to inhibit podocyte apoptosis, while the suppression of microRNA-20b promotes SIRT7 expression to decrease apoptosis (137) (**Table 9**). This research demonstrated that increasing the expression of SIRT7 reduced the occurrence of apoptosis in podocytes.

**Table 9:** DKD studied on other pathways.

Reference	Drug/Target	Sirtuins	Model	Mechanism of protection	Pathway
(24)	SIRT3	Reduction of SIRT3 activity	ZDF rat T2DM model HG-induced HK-2	Enhance mitochondrial oxidative stress	CD38 OE, intracellular NAD^+^/NADH ratio
(28)	SIRT4 OE	SIRT4	HG-induced mouse podocytes	Attenuate inflammatory response, prevent apoptosis and ROS production	Inhibit apoptosis via the mitochondrial pathway
(30)	SIRT6 OE	Increase SIRT6 expression	STZ rats Mouse podocyte MPC-5	Promote M2 macrophage transformation, alleviate renal injury	Upregulate the expression of Bcl−2 and CD206, and decrease expression of Bax and CD86
(31)	SIRT6 OE	Increase SIRT6 expression	STZ-induced C57BL/6 mouse, db/db mouse AGE/HG induced human podocytes	Anti-apoptosis and -inflammation by increasing autophagic flux	Through inhibition of the Notch pathway
(123)	1α,25-Dihydroxyvitamin D3	Activate SIRT1	ZDF rats	Antioxidant	PARP1/SIRT1/ NOX4 pathway
(124)	Puerarin	Increase SIRT1 expression	STZ-induced eNOS-null C57BL/6 male mouse HG-induced murine podocytes	Anti-oxidative	Through the suppression of NOX4 expression
(125)	Carnosine	Upregulation of SIRT1	HG-induced podocyte	Reduce glycative and lipoperoxidative stress.	Hsp70, SIRT1, Trx, γ-GCS, HO-1
(126)	Anserine	No effect on SIRT1	db/db mouse HG-induced HK-2	Anti-oxidant and glycative stress	Hsp70/HO-1 defense system
(127)	Inhibition of HIC1	Rescue SIRT1 expression	HG-induced HK-2	Reduce ROS accumulation	Target the HIC1/EZH2/DNMT1 axis
(128)	INT-767	Restore SIRT1 expression	STZ-induced DBA/2J mouse, db/db mice with T2DM	Prevent inflammation, oxidative stress, endoplasmic reticulum stress, and tubulointerstitial fibrosis	Induce mitochondrial biogenesis pathway, prevents activation of pofibrotic signaling pathways
(129)	SGLT2 inhibition	Restore SIRT1	Male C57BL/6 db/db mouse HG-cultured porcine LLC-PK1 cells	Prevent intracellular glucose entry from the apical side into the proximal tubular cells	GLUT2/importin-α1/HNF-1α pathway
(130)	Aerobic exercise training	Restore SIRT1 expression	STZ induced C57BL/6 mouse T1DM	Improve mitochondrial function	MMP, ATP, superoxide production
(131)	Apigenin	Increase SIRT3 activity	Male diabetic fatty rats HG-induced HK-2 cells	Relieve mitochondrial oxidative stress	Restore the intracellular NAD^+^/NADH ratio and SIRT3 activity
(132)	Empagliflozin	Restore SIRT3 levels	STZ mice HG-induced HK-2	Suppress the EMT, with restoration of all aberrant functions	Inhibiting glucose uptake into the proximal tubule
(133)	Liraglutide	Upregulate SIRT3 expression	HG induced HRMCs	Prevent activation of mitochondrial apoptosis	Activate ERK/Yap signaling pathway
(134)	INT-777	Increase activity of SIRT1 and SIRT3	db/db diabetic mouse	Increase mitochondrial biogenesis, decrease oxidative stress and fibrosis	TGR5 signaling
(135)	AFSCs transplantation	SIRT3 OE in AFSCs	C57BL/KsJ db/db mouse	Reduce apoptosis and fibrosis	By modulation of mitophagy
(136)	Nampt	SIRT6	STZ induced male mouse HK-2	Reduce fibrogenic extracellular matrix remodeling	Nampt/SIRT6 axis
(137)	Suppression of microRNA-20b	SIRT7 OE	HG−induced podocyte	Inhibit the podocyte apoptosis	By targeting SIRT7
(148)	AGEs-RAGE system	Down-regulate SIRT1	AGEs-induced GMCs	Diabetic renal fibrosis	Through the ubiquitin-proteasome pathway
(149)	Resveratrol	Restore SIRT1 expression	STZ-induced SD rat as a T1DM model HG-induced mouse podocytes	Modulate angiogenesis, reduce GBM thickness and fibrosis	Via modulating the angiogenic factors

### 5.9 Summary of SIRT1–SIRT7

**Figure 2:**
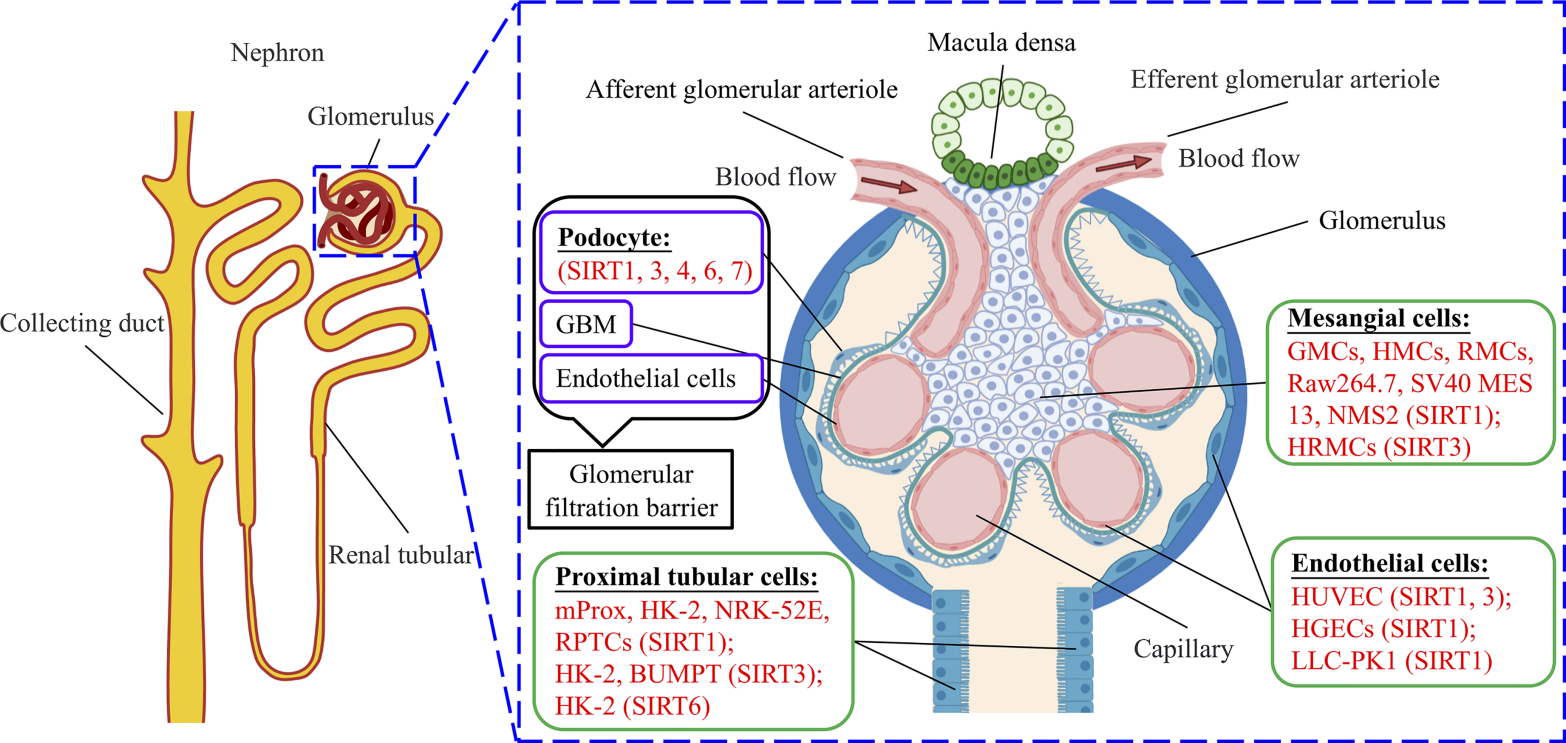
Basic structure of nephron and glomerulus and different cell models for sirtuins studies.

SIRT1 was the first sirtuin discovered in mammals, and remains the most extensively and deeply studied so far (138). Resveratrol is the most recognized and studied activator of SIRT1 (139). SIRT1 has been extensively studied in DKD models, including podocytes, mesangial cells, and tubular cells. SIRT2 is the only cytoplasmic sirtuin, but its role in treating DKD has not been reported yet so far, nor has that of SIRT5. SIRT3 is normally located in the mitochondria, but under cellular stress, it can translocate into the nucleus (140). Some studies have reported that increased expression of SIRT3 is beneficial to DKD, mainly through AMPK or PI3K pathways (25, 27, 54, 109, 110). However, we found one article that reported that the overexpression of SIRT4 reduced inflammatory effects, and inhibited ROS production and apoptosis in HG-induced podocytes (28). SIRT6 is a nuclear HDAC that plays an important role in the pathological processes of inflammation, aging, cancer, and neurodegenerative diseases (141). However, only a few studies on SIRT6 have been reported, mainly in podocyte and tubular cell models of DKD. Additionally, the catalytic activity of SIRT7 is weak, and a previous report indicated that the suppression of microRNA-20b increased SIRT7 expression and reduced HG-induced podocyte apoptosis (137) (**Figure 2**).

## 6 Conclusions and Perspectives

**Figure 3:**
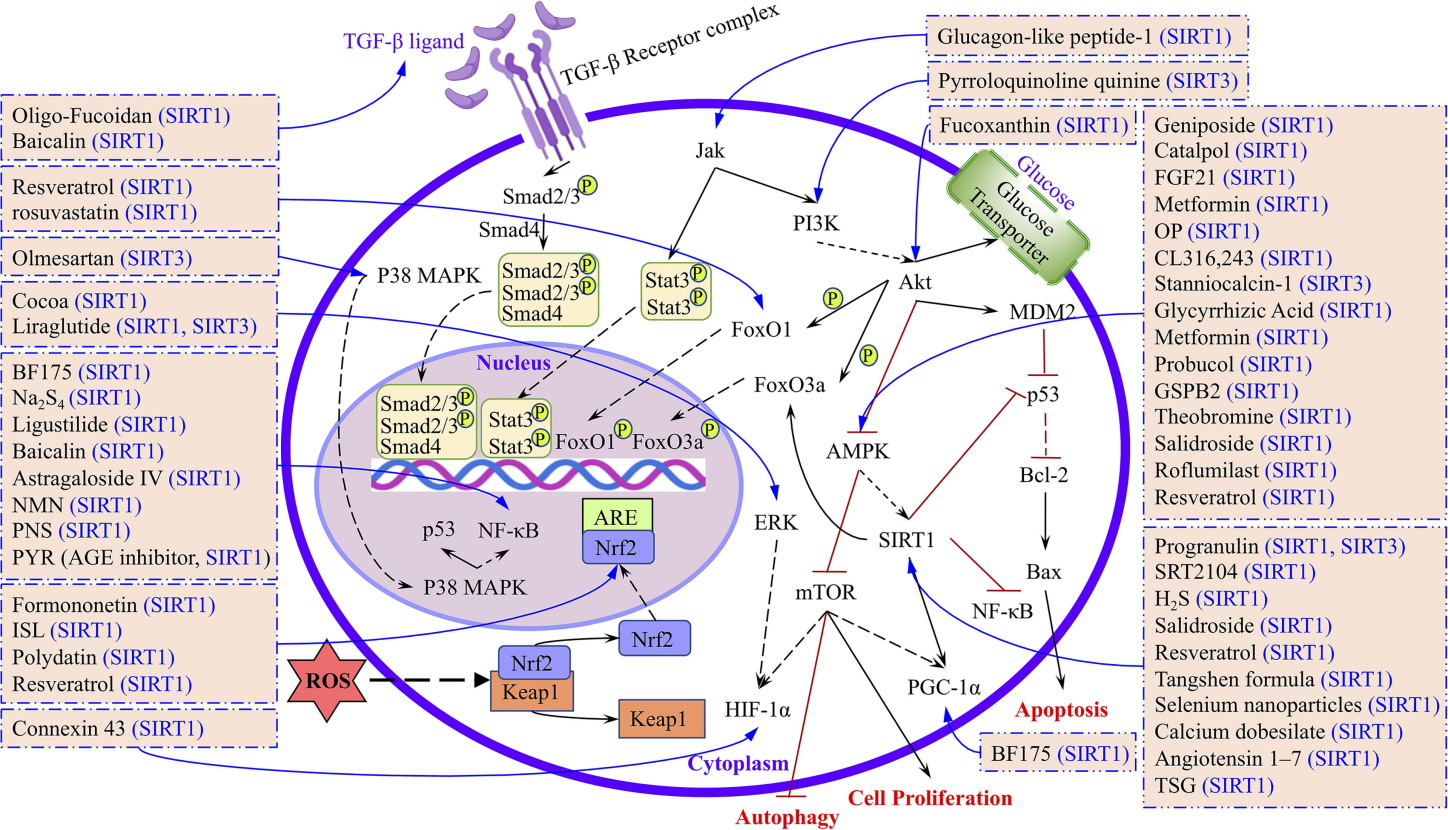
The targets and signaling pathways of different drugs or compounds regulated by sirtuins were summarized.

Many researchers are working to investigate the etiology of DKD and explore new treatment methods. In our conventional view, sirtuins are a class of HDACs involved in the regulation of longevity and maintaining the stability of nucleosomes by balancing with histone acetylases (13). However, in addition to deacetylate histones, we discovered that sirtuins also regulate many transcription factors, including FOXO1, FOXO3a, STAT3, Smad2/3, NF-κB, p53, and Nrf-2. These transcription factors are involved in regulating many biological processes, including autophagy, oxidative stress, apoptosis, inflammation, EMT, and fibrosis (**Figure 3**). We found that in DKD studies, the high expression of SIRT1–SIRT7 alleviated or reduced kidney injury through different mechanisms or molecular pathways, of which SIRT1 is the most widely explored. However, an exception was found in db/db mice, which showed that treatment with glucagon-like peptide-1 reduced SIRT1 expression, while in HUVEC cells, glucagon-like peptide-1 had no significant effect on the SIRT1 expression level. The authors explained that the *in vivo* results were due to a reduced inflammatory environment that did not stimulate SIRT1, while the *in vitro* results were due to SIRT1 only participating in transcriptional responses (122). Resveratrol is a recognized activator of SIRT1, but in db/db mice, treatment with resveratrol failed to cause changes in SIRT1 expression, and it still improved oxidative stress and enhanced mitochondrial biogenesis in the AMPK/SIRT1-independent pathway (59). Furthermore, the expression of *SIRT1*, *SIRT2*, *SIRT3*, and *SIRT6* was higher than *SIRT4*, *SIRT5*, and *SIRT7* in the kidney; therefore, the study of SIRT1, SIRT2, SIRT3, and SIRT6 in DKD models is both reasonable and credible (136).

In light of the above, to better illuminate the roles of SIRT1–SIRT7 in DKD and the research progress, we have summarized the therapeutics, targets, and signaling pathways involved in *in vitro* and *in vivo* models of DKD (**Figure 3**). Our aim is that this review will serve as a valuable reference for future studies of sirtuins and DKD, and provide a theoretical foundation for delaying the pathological process of DKD in the clinic.

## Author Contributions

WQ and CH contributed to designing and writing the manuscript. DZ and XL approved the submitted version. All authors contributed to the article and approved the submitted version.

## Funding

This work was supported by the National Natural Science Foundation of China (U19A2013), the National Key Research and Development Program of China (2017YFC1702103), and the Science and Technology Development Plan Project of Jilin Province (202002053JC).

## Conflict of Interest

The authors declare that the research was conducted in the absence of any commercial or financial relationships that could be construed as a potential conflict of interest.

## Publisher’s Note

All claims expressed in this article are solely those of the authors and do not necessarily represent those of their affiliated organizations, or those of the publisher, the editors and the reviewers. Any product that may be evaluated in this article, or claim that may be made by its manufacturer, is not guaranteed or endorsed by the publisher.

## Abbreviations

AFSCs, Amniotic fluid stem cells; AGEs, Advanced glycation end products; BAT, Brown adipose tissue; EMT, Epithelial-mesenchymal transition; GBM, Glomerular basement membrane; GMCs, Glomerular mesangial cells; GSPB2, Grape seed procyanidin B2; HFD, High-fat diet; HG, High glucose; HGECs, Human glomerular endothelial cells; HIC1, Hypermethylated in cancer 1; hnRNP F, Heterogeneous Nuclear Ribonucleoprotein F; IRPTCs, ROS generation mediates HG stimulation of angiotensinogen expression in immortalized rat RPT cells; ISLQ, Isoliquiritigenin; KD, Knockdown; Nampt, NMN-producing enzyme nicotinamide phosphoribosyltransferase; NMN, Nicotinamide mononucleotide; OE, Overexpression; OP, A polysaccharide purified from okra; OLETF, Otsuka-Long-Evans-Tokushima-Fatty; PNS, Panax notoginseng saponins; RMCs, Renal mesangial cells; ROS, Reactive oxygen species; STZ, Streptozotocin; T1DM, Type 1 diabetes mellitus; T2DM, Type 2 diabetes mellitus; TSG, Tetrahydroxystilbene glucoside.

## References

[1] Wang AJ Wang S Wang BJ Xiao M Guo Y Tang Y. Epigenetic Regulation Associated With Sirtuin 1 in Complications of Diabetes Mellitus. Front Endocrinol (Lausanne) (2020) 11:598012. doi: 10.3389/fendo.2020.598012 33537003 PMC7848207

[2] Hershberger KA Martin AS Hirschey MD. Role of NAD(+) and Mitochondrial Sirtuins in Cardiac and Renal Diseases. Nat Rev Nephrol (2017) 13(4):213–25. doi: 10.1038/nrneph.2017.5 PMC5508210 28163307

[3] Wakino S Hasegawa K Itoh H. Sirtuin and Metabolic Kidney Disease. Kidney Int (2015) 88(4):691–8. doi: 10.1038/ki.2015.157 PMC4593995 26083654

[4] Navarro-Gonzalez JF Mora-Fernandez C Muros de Fuentes M Garcia-Perez J. Inflammatory Molecules and Pathways in the Pathogenesis of Diabetic Nephropathy. Nat Rev Nephrol (2011) 7(6):327–40. doi: 10.1038/nrneph.2011.51 21537349

[5] Gheith O Farouk N Nampoory N Halim MA Al-Otaibi T. Diabetic Kidney Disease: World Wide Difference of Prevalence and Risk Factors. J Nephropharmacol (2016) 5(1):49–56. doi: 10.4103/1110-9165.197379 28197499 PMC5297507

[6] Koye DN Magliano DJ Nelson RG Pavkov ME. The Global Epidemiology of Diabetes and Kidney Disease. Adv Chronic Kidney Dis (2018) 25(2):121–32. doi: 10.1053/j.ackd.2017.10.011 PMC11000253 29580576

[7] Wang W Sun W Cheng Y Xu Z Cai L. Role of Sirtuin-1 in Diabetic Nephropathy. J Mol Med (Berl) (2019) 97(3):291–309. doi: 10.1007/s00109-019-01743-7 30707256 PMC6394539

[8] Li X Zhang J Xie Y Jiang Y Yingjie Z Xu W. Progress of HDAC Inhibitor Panobinostat in the Treatment of Cancer. Curr Drug Targets (2014) 15(6):622–34. doi: 10.2174/1389450115666140306152642 24597570

[9] Chen B Zang W Wang J Huang Y He Y Yan L. The Chemical Biology of Sirtuins. Chem Soc Rev (2015) 44(15):5246–64. doi: 10.1039/c4cs00373j 25955411

[10] Morigi M Perico L Benigni A. Sirtuins in Renal Health and Disease. J Am Soc Nephrol (2018) 29(7):1799–809. doi: 10.1681/ASN.2017111218 PMC6050939 29712732

[11] Wang Y He J Liao M Hu M Li W Ouyang H. An Overview of Sirtuins as Potential Therapeutic Target: Structure, Function and Modulators. Eur J Med Chem (2019) 161:48–77. doi: 10.1016/j.ejmech.2018.10.028 30342425

[12] Yoon YK Oon CE. Sirtuin Inhibitors: An Overview From Medicinal Chemistry Perspective. Anticancer Agents Med Chem (2016) 16(8):1003–16. doi: 10.2174/1871520616666160310141622 26961318

[13] Watroba M Dudek I Skoda M Stangret A Rzodkiewicz P Szukiewicz D. Sirtuins, Epigenetics and Longevity. Ageing Res Rev (2017) 40:11–9. doi: 10.1016/j.arr.2017.08.001 28789901

[14] Leite JA Ghirotto B Targhetta VP de Lima J Camara NOS. Sirtuins as Pharmacological Targets in Neurodegenerative and Neuropsychiatric Disorders. Br J Pharmacol (2021) 179(8):1496–511. doi: 10.1111/bph.15570 34029375

[15] Zhu S Dong Z Ke X Hou J Zhao E Zhang K. The Roles of Sirtuins Family in Cell Metabolism During Tumor Development. Semin Cancer Biol (2019) 57:59–71. doi: 10.1016/j.semcancer.2018.11.003 30453040

[16] van de Ven RAH Santos D Haigis MC. Mitochondrial Sirtuins and Molecular Mechanisms of Aging. Trends Mol Med (2017) 23(4):320–31. doi: 10.1016/j.molmed.2017.02.005 PMC5713479 28285806

[17] DeFronzo RA Reeves WB Awad AS. Pathophysiology of Diabetic Kidney Disease: Impact of SGLT2 Inhibitors. Nat Rev Nephrol (2021) 17(5):319–34. doi: 10.1038/s41581-021-00393-8 33547417

[18] Zoja C Xinaris C Macconi D. Diabetic Nephropathy: Novel Molecular Mechanisms and Therapeutic Targets. Front Pharmacol (2020) 11:586892. doi: 10.3389/fphar.2020.586892 33519447 PMC7845653

[19] Gnudi L Coward RJM Long DA. Diabetic Nephropathy: Perspective on Novel Molecular Mechanisms. Trends Endocrinol Metab (2016) 27(11):820–30. doi: 10.1016/j.tem.2016.07.002 27470431

[20] Liu S Yuan Y Xue Y Xing C Zhang B. Podocyte Injury in Diabetic Kidney Disease: A Focus on Mitochondrial Dysfunction. Front Cell Dev Biol (2022) 10:832887. doi: 10.3389/fcell.2022.832887 35321238 PMC8935076

[21] Chuang PY Xu J Dai Y Jia F Mallipattu SK Yacoub R. *In Vivo* RNA Interference Models of Inducible and Reversible Sirt1 Knockdown in Kidney Cells. Am J Pathol (2014) 184(7):1940–56. doi: 10.1016/j.ajpath.2014.03.016 PMC4076473 24952428

[22] Huang K Gao X Wei W. The Crosstalk Between Sirt1 and Keap1/Nrf2/ARE Anti-Oxidative Pathway Forms a Positive Feedback Loop to Inhibit FN and TGF-Beta1 Expressions in Rat Glomerular Mesangial Cells. Exp Cell Res (2017) 361(1):63–72. doi: 10.1016/j.yexcr.2017.09.042 28986066

[23] Zhou L Xu DY Sha WG Shen L Lu GY Yin X. High Glucose Induces Renal Tubular Epithelial Injury *via* Sirt1/NF-Kappab/microR-29/Keap1 Signal Pathway. J Transl Med (2015) 13:352. doi: 10.1186/s12967-015-0710-y 26552447 PMC4640239

[24] Ogura Y Kitada M Monno I Kanasaki K Watanabe A Koya D. Renal Mitochondrial Oxidative Stress is Enhanced by the Reduction of Sirt3 Activity, in Zucker Diabetic Fatty Rats. Redox Rep (2018) 23(1):153–9. doi: 10.1080/13510002.2018.1487174 PMC6748695 29897845

[25] Jiao X Li Y Zhang T Liu M Chi Y. Role of Sirtuin3 in High Glucose-Induced Apoptosis in Renal Tubular Epithelial Cells. Biochem Biophys Res Commun (2016) 480(3):387–93. doi: 10.1016/j.bbrc.2016.10.060 27773814

[26] Srivastava SP Li J Kitada M Fujita H Yamada Y Goodwin JE. SIRT3 Deficiency Leads to Induction of Abnormal Glycolysis in Diabetic Kidney With Fibrosis. Cell Death Dis (2018) 9(10):997. doi: 10.1038/s41419-018-1057-0 30250024 PMC6155322

[27] Wang Y Zhang X Wang P Shen Y Yuan K Li M. Sirt3 Overexpression Alleviates Hyperglycemia-Induced Vascular Inflammation Through Regulating Redox Balance, Cell Survival, and AMPK-Mediated Mitochondrial Homeostasis. J Recept Signal Transduct Res (2019) 39(4):341–9. doi: 10.1080/10799893.2019.1684521 31680596

[28] Shi JX Wang QJ Li H Huang Q. SIRT4 Overexpression Protects Against Diabetic Nephropathy by Inhibiting Podocyte Apoptosis. Exp Ther Med (2017) 13(1):342–8. doi: 10.3892/etm.2016.3938 PMC5245066 28123512

[29] Fan Y Yang Q Yang Y Gao Z Ma Y Zhang L. Sirt6 Suppresses High Glucose-Induced Mitochondrial Dysfunction and Apoptosis in Podocytes Through AMPK Activation. Int J Biol Sci (2019) 15(3):701–13. doi: 10.7150/ijbs.29323 PMC6367578 30745856

[30] Ji L Chen Y Wang H Zhang W He L Wu J. Overexpression of Sirt6 Promotes M2 Macrophage Transformation, Alleviating Renal Injury in Diabetic Nephropathy. Int J Oncol (2019) 55(1):103–15. doi: 10.3892/ijo.2019.4800 PMC6561622 31115579

[31] Liu M Liang K Zhen J Zhou M Wang X Wang Z. Sirt6 Deficiency Exacerbates Podocyte Injury and Proteinuria Through Targeting Notch Signaling. Nat Commun (2017) 8(1):413. doi: 10.1038/s41467-017-00498-4 28871079 PMC5583183

[32] Abbas S Raza ST Ahmed F Ahmad A Rizvi S Mahdi F. Association of Genetic Polymorphism of PPARgamma-2, ACE, MTHFR, FABP-2 and FTO Genes in Risk Prediction of Type 2 Diabetes Mellitus. J BioMed Sci (2013) 20:80. doi: 10.1186/1423-0127-20-80 24156506 PMC4015124

[33] Li YY. ENPP1 K121Q Polymorphism and Type 2 Diabetes Mellitus in the Chinese Population: A Meta-Analysis Including 11,855 Subjects. Metabolism (2012) 61(5):625–33. doi: 10.1016/j.metabol.2011.10.002 22136912

[34] Dobigny G Britton-Davidian J Robinson TJ. Chromosomal Polymorphism in Mammals: An Evolutionary Perspective. Biol Rev Camb Philos Soc (2017) 92(1):1–21. doi: 10.1111/brv.12213 26234165

[35] Zhao Y Wei J Hou X Liu H Guo F Zhou Y. SIRT1 Rs10823108 and FOXO1 Rs17446614 Responsible for Genetic Susceptibility to Diabetic Nephropathy. Sci Rep (2017) 7(1):10285. doi: 10.1038/s41598-017-10612-7 28860538 PMC5579017

[36] Tang K Sun M Shen J Zhou B. Transcriptional Coactivator P300 and Silent Information Regulator 1 (SIRT1) Gene Polymorphism Associated With Diabetic Kidney Disease in a Chinese Cohort. Exp Clin Endocrinol Diabetes (2017) 125(8):530–7. doi: 10.1055/s-0043-103966 28444663

[37] Ren H Shao Y Ma X Yang M Liu Y Wang Q. Expression Levels of Serum Vasohibin-1 and Other Biomarkers in Type 2 Diabetes Mellitus Patients With Different Urinary Albumin to Creatinine Ratios. J Diabetes Complications (2019) 33(7):477–84. doi: 10.1016/j.jdiacomp.2019.04.008 31097304

[38] Yubero-Serrano EM Woodward M Poretsky L Vlassara H Striker GE Group AG-lS. Effects of Sevelamer Carbonate on Advanced Glycation End Products and Antioxidant/Pro-Oxidant Status in Patients With Diabetic Kidney Disease. Clin J Am Soc Nephrol (2015) 10(5):759–66. doi: 10.2215/CJN.07750814 PMC4422240 25710801

[39] Bian C Gao J Wang Y Li J Luan Z Lu H. Association of SIRT6 Circulating Levels With Urinary and Glycometabolic Markers in Pre-Diabetes and Diabetes. Acta Diabetol (2021) 58(11):1551–62. doi: 10.1007/s00592-021-01759-x 34148121

[40] Akhtar S Siragy HM. Pro-Renin Receptor Suppresses Mitochondrial Biogenesis and Function *via* AMPK/SIRT-1/ PGC-1alpha Pathway in Diabetic Kidney. PloS One (2019) 14(12):e0225728. doi: 10.1371/journal.pone.0225728 31800607 PMC6892478

[41] Kim MY Lim JH Youn HH Hong YA Yang KS Park HS. Resveratrol Prevents Renal Lipotoxicity and Inhibits Mesangial Cell Glucotoxicity in a Manner Dependent on the AMPK-SIRT1-PGC1alpha Axis in Db/Db Mice. Diabetologia (2013) 56(1):204–17. doi: 10.1007/s00125-012-2747-2 23090186

[42] Liao Z Zhang J Wang J Yan T Xu F Wu B. The Anti-Nephritic Activity of a Polysaccharide From Okra (Abelmoschus Esculentus (L.) Moench) *via* Modulation of AMPK-Sirt1-PGC-1alpha Signaling Axis Mediated Anti-Oxidative in Type 2 Diabetes Model Mice. Int J Biol Macromol (2019) 140:568–76. doi: 10.1016/j.ijbiomac.2019.08.149 31442509

[43] Cai YY Zhang HB Fan CX Zeng YM Zou SZ Wu CY. Renoprotective Effects of Brown Adipose Tissue Activation in Diabetic Mice. J Diabetes (2019) 11(12):958–70. doi: 10.1111/1753-0407.12938 PMC6899899 31020790

[44] Hou S Zhang T Li Y Guo F Jin X. Glycyrrhizic Acid Prevents Diabetic Nephropathy by Activating AMPK/SIRT1/PGC-1alpha Signaling in Db/Db Mice. J Diabetes Res (2017) 2017:2865912. doi: 10.1155/2017/2865912 29238727 PMC5697128

[45] Park HS Lim JH Kim MY Kim Y Hong YA Choi SR. Resveratrol Increases AdipoR1 and AdipoR2 Expression in Type 2 Diabetic Nephropathy. J Transl Med (2016) 14(1):176. doi: 10.1186/s12967-016-0922-9 27286657 PMC4902973

[46] Cai X Bao L Ren J Li Y Zhang Z. Grape Seed Procyanidin B2 Protects Podocytes From High Glucose-Induced Mitochondrial Dysfunction and Apoptosis *via* the AMPK-SIRT1-PGC-1alpha Axis *In Vitro* . Food Funct (2016) 7(2):805–15. doi: 10.1039/c5fo01062d 26650960

[47] Bao L Cai X Zhang Z Li Y. Grape Seed Procyanidin B2 Ameliorates Mitochondrial Dysfunction and Inhibits Apoptosis *via* the AMP-Activated Protein Kinase-Silent Mating Type Information Regulation 2 Homologue 1-PPARgamma Co-Activator-1alpha Axis in Rat Mesangial Cells Under High-Dose Glucosamine. Br J Nutr (2015) 113(1):35–44. doi: 10.1017/S000711451400347X 25404010

[48] Weng W Ge T Wang Y He L Liu T Wang W. Therapeutic Effects of Fibroblast Growth Factor-21 on Diabetic Nephropathy and the Possible Mechanism in Type 1 Diabetes Mellitus Mice. Diabetes Metab J (2020) 44(4):566–80. doi: 10.4093/dmj.2019.0089 PMC7453991 32431116

[49] Rogacka D Audzeyenka I Rychlowski M Rachubik P Szrejder M Angielski S. Metformin Overcomes High Glucose-Induced Insulin Resistance of Podocytes by Pleiotropic Effects on SIRT1 and AMPK. Biochim Biophys Acta Mol Basis Dis (2018) 1864(1):115–25. doi: 10.1016/j.bbadis.2017.10.014 29032153

[50] Shati AA. Salidroside Ameliorates Diabetic Nephropathy in Rats by Activating Renal AMPK/SIRT1 Signaling Pathway. J Food Biochem (2020) 44(4):e13158. doi: 10.1111/jfbc.13158 32030786

[51] Tikoo K Lodea S Karpe PA Kumar S. Calorie Restriction Mimicking Effects of Roflumilast Prevents Diabetic Nephropathy. Biochem Biophys Res Commun (2014) 450(4):1581–6. doi: 10.1016/j.bbrc.2014.07.039 25035926

[52] Chen J Yang Y Lv Z Shu A Du Q Wang W. Study on the Inhibitive Effect of Catalpol on Diabetic Nephropathy. Life Sci (2020) 257:118120. doi: 10.1016/j.lfs.2020.118120 32693244

[53] Li F Chen Y Li Y Huang M Zhao W. Geniposide Alleviates Diabetic Nephropathy of Mice Through AMPK/SIRT1/NF-kappaB Pathway. Eur J Pharmacol (2020) 886:173449. doi: 10.1016/j.ejphar.2020.173449 32758570

[54] Liu Z Liu H Xiao L Liu G Sun L He L. STC-1 Ameliorates Renal Injury in Diabetic Nephropathy by Inhibiting the Expression of BNIP3 Through the AMPK/SIRT3 Pathway. Lab Invest (2019) 99(5):684–97. doi: 10.1038/s41374-018-0176-7 30683904

[55] Ren H Shao Y Wu C Ma X Lv C Wang Q. Metformin Alleviates Oxidative Stress and Enhances Autophagy in Diabetic Kidney Disease *via* AMPK/SIRT1-FoxO1 Pathway. Mol Cell Endocrinol (2020) 500:110628. doi: 10.1016/j.mce.2019.110628 31647955

[56] Alvarez-Cilleros D Lopez-Oliva ME Martin MA Ramos S. Cocoa Ameliorates Renal Injury in Zucker Diabetic Fatty Rats by Preventing Oxidative Stress, Apoptosis and Inactivation of Autophagy. Food Funct (2019) 10(12):7926–39. doi: 10.1039/c9fo01806a 31773121

[57] Yang S Zhao L Han Y Liu Y Chen C Zhan M. Probucol Ameliorates Renal Injury in Diabetic Nephropathy by Inhibiting the Expression of the Redox Enzyme p66Shc. Redox Biol (2017) 13:482–97. doi: 10.1016/j.redox.2017.07.002 PMC5514499 28728079

[58] Hou S Zheng F Li Y Gao L Zhang J. The Protective Effect of Glycyrrhizic Acid on Renal Tubular Epithelial Cell Injury Induced by High Glucose. Int J Mol Sci (2014) 15(9):15026–43. doi: 10.3390/ijms150915026 PMC4200778 25162824

[59] Kitada M Kume S Imaizumi N Koya D. Resveratrol Improves Oxidative Stress and Protects Against Diabetic Nephropathy Through Normalization of Mn-SOD Dysfunction in AMPK/SIRT1-Independent Pathway. Diabetes (2011) 60(2):634–43. doi: 10.2337/db10-0386 PMC3028365 21270273

[60] Papadimitriou A Silva KC Peixoto EB Borges CM Lopes de Faria JM Lopes de Faria JB. Theobromine Increases NAD(+)/Sirt-1 Activity and Protects the Kidney Under Diabetic Conditions. Am J Physiol Renal Physiol (2015) 308(3):F209–25. doi: 10.1152/ajprenal.00252.2014 25411384

[61] Locatelli M Zoja C Zanchi C Corna D Villa S Bolognini S. Manipulating Sirtuin 3 Pathway Ameliorates Renal Damage in Experimental Diabetes. Sci Rep (2020) 10(1):8418. doi: 10.1038/s41598-020-65423-0 32439965 PMC7242337

[62] Zhang T Chi Y Kang Y Lu H Niu H Liu W. Resveratrol Ameliorates Podocyte Damage in Diabetic Mice *via* SIRT1/PGC-1alpha Mediated Attenuation of Mitochondrial Oxidative Stress. J Cell Physiol (2019) 234(4):5033–43. doi: 10.1002/jcp.27306 30187480

[63] Xue H Li P Luo Y Wu C Liu Y Qin X. Salidroside Stimulates the Sirt1/PGC-1alpha Axis and Ameliorates Diabetic Nephropathy in Mice. Phytomedicine (2019) 54:240–7. doi: 10.1016/j.phymed.2018.10.031 30668374

[64] Hong Q Zhang L Das B Li Z Liu B Cai G. Increased Podocyte Sirtuin-1 Function Attenuates Diabetic Kidney Injury. Kidney Int (2018) 93(6):1330–43. doi: 10.1016/j.kint.2017.12.008 PMC5967974 29477240

[65] Oza MJ Kulkarni YA. Formononetin Attenuates Kidney Damage in Type 2 Diabetic Rats. Life Sci (2019) 219:109–21. doi: 10.1016/j.lfs.2019.01.013 30641085

[66] Shi JX Huang Q. Glucagonlike Peptide1 Protects Mouse Podocytes Against High Glucoseinduced Apoptosis, and Suppresses Reactive Oxygen Species Production and Proinflammatory Cytokine Secretion, Through Sirtuin 1 Activation *In Vitro* . Mol Med Rep (2018) 18(2):1789–97. doi: 10.3892/mmr.2018.9085 29845208

[67] Ma L Fu R Duan Z Lu J Gao J Tian L. Sirt1 is Essential for Resveratrol Enhancement of Hypoxia-Induced Autophagy in the Type 2 Diabetic Nephropathy Rat. Pathol Res Pract (2016) 212(4):310–8. doi: 10.1016/j.prp.2016.02.001 26872534

[68] Ge X Xu B Xu W Xia L Xu Z Shen L. Long Noncoding RNA GAS5 Inhibits Cell Proliferation and Fibrosis in Diabetic Nephropathy by Sponging miR-221 and Modulating SIRT1 Expression. Aging (Albany NY) (2019) 11(20):8745–59. doi: 10.18632/aging.102249 PMC6834398 31631065

[69] Zhang Y Chang B Zhang J Wu X. LncRNA SOX2OT Alleviates the High Glucose-Induced Podocytes Injury Through Autophagy Induction by the miR-9/SIRT1 Axis. Exp Mol Pathol (2019) 110:104283. doi: 10.1016/j.yexmp.2019.104283 31301307

[70] Liu F Guo J Qiao Y Pan S Duan J Liu D. MiR-138 Plays an Important Role in Diabetic Nephropathy Through SIRT1-P38-TTP Regulatory Axis. J Cell Physiol (2021) 236(9):6607–18. doi: 10.1002/jcp.30238 33843045

[71] Smith J. Human Sir2 and the 'Silencing' of P53 Activity. Trends Cell Biol (2002) 12(9):404–6. doi: 10.1016/s0962-8924(02)02342-5 12220851

[72] Cohen HY Miller C Bitterman KJ Wall NR Hekking B Kessler B. Calorie Restriction Promotes Mammalian Cell Survival by Inducing the SIRT1 Deacetylase. Science (2004) 305(5682):390–2. doi: 10.1126/science.1099196 15205477

[73] Wang Y Zheng ZJ Jia YJ Yang YL Xue YM. Role of P53/miR-155-5p/Sirt1 Loop in Renal Tubular Injury of Diabetic Kidney Disease. J Transl Med (2018) 16(1):146. doi: 10.1186/s12967-018-1486-7 29848325 PMC5975703

[74] Dong W Zhang H Zhao C Luo Y Chen Y. Silencing of miR-150-5p Ameliorates Diabetic Nephropathy by Targeting SIRT1/p53/AMPK Pathway. Front Physiol (2021) 12:624989. doi: 10.3389/fphys.2021.624989 33897448 PMC8064124

[75] Ahmed HH Taha FM Omar HS Elwi HM Abdelnasser M. Hydrogen Sulfide Modulates SIRT1 and Suppresses Oxidative Stress in Diabetic Nephropathy. Mol Cell Biochem (2019) 457(1-2):1–9. doi: 10.1007/s11010-019-03506-x 30778838

[76] Wang XL Wu LY Zhao L Sun LN Liu HY Liu G. SIRT1 Activator Ameliorates the Renal Tubular Injury Induced by Hyperglycemia *In Vivo* and *In Vitro via* Inhibiting Apoptosis. BioMed Pharmacother (2016) 83:41–50. doi: 10.1016/j.biopha.2016.06.009 27470548

[77] Wang Y Zuo B Wang N Li S Liu C Sun D. Calcium Dobesilate Mediates Renal Interstitial Fibrosis and Delay Renal Peritubular Capillary Loss Through Sirt1/p53 Signaling Pathway. BioMed Pharmacother (2020) 132:110798. doi: 10.1016/j.biopha.2020.110798 33011612

[78] Deng Z Jin J Wang Z Wang Y Gao Q Zhao J. The Metal Nanoparticle-Induced Inflammatory Response is Regulated by SIRT1 Through NF-kappaB Deacetylation in Aseptic Loosening. Int J Nanomed (2017) 12:3617–36. doi: 10.2147/IJN.S124661 PMC5439723 28553103

[79] Nopparat C Sinjanakhom P Govitrapong P. Melatonin Reverses H2 O2 -Induced Senescence in SH-SY5Y Cells by Enhancing Autophagy *via* Sirtuin 1 Deacetylation of the RelA/p65 Subunit of NF-Kappab. J Pineal Res (2017) 63(1):1–13. doi: 10.1111/jpi.12407 28295567

[80] Li J Ling Y Yin S Yang S Kong M Li Z. Baicalin Serves a Protective Role in Diabetic Nephropathy Through Preventing High Glucose-Induced Podocyte Apoptosis. Exp Ther Med (2020) 20(1):367–74. doi: 10.3892/etm.2020.8701 PMC7296293 32550886

[81] Alzahrani S Zaitone SA Said E El-Sherbiny M Ajwah S Alsharif SY. Protective Effect of Isoliquiritigenin on Experimental Diabetic Nephropathy in Rats: Impact on Sirt-1/NFkappaB Balance and NLRP3 Expression. Int Immunopharmacol (2020) 87:106813. doi: 10.1016/j.intimp.2020.106813 32707499

[82] Wang X Gao Y Tian N Wang T Shi Y Xu J. Astragaloside IV Inhibits Glucose-Induced Epithelial-Mesenchymal Transition of Podocytes Through Autophagy Enhancement *via* the SIRT-NF-kappaB P65 Axis. Sci Rep (2019) 9(1):323. doi: 10.1038/s41598-018-36911-1 30674969 PMC6344540

[83] Du YG Zhang KN Gao ZL Dai F Wu XX Chai KF. Tangshen Formula Improves Inflammation in Renal Tissue of Diabetic Nephropathy Through SIRT1/NF-kappaB Pathway. Exp Ther Med (2018) 15(2):2156–64. doi: 10.3892/etm.2017.5621 PMC5776509 29434819

[84] Chen Y Liang Y Hu T Wei R Cai C Wang P. Endogenous Nampt Upregulation is Associated With Diabetic Nephropathy Inflammatory-Fibrosis Through the NF-kappaB P65 and Sirt1 Pathway; NMN Alleviates Diabetic Nephropathy Inflammatory-Fibrosis by Inhibiting Endogenous Nampt. Exp Ther Med (2017) 14(5):4181–93. doi: 10.3892/etm.2017.5098 PMC5658765 29104634

[85] Wang X Gao Y Tian N Zhu Z Wang T Xu J. Astragaloside IV Represses High Glucose-Induced Mesangial Cells Activation by Enhancing Autophagy *via* SIRT1 Deacetylation of NF-kappaB P65 Subunit. Drug Des Devel Ther (2018) 12:2971–80. doi: 10.2147/DDDT.S174058 PMC6140761 30254426

[86] Xu F Ye Z Tao S Liu W Su J Fang X. Ligustilide Alleviates Podocyte Injury *via* Suppressing the SIRT1/NF-kappaB Signaling Pathways in Rats With Diabetic Nephropathy. Ann Transl Med (2020) 8(18):1154. doi: 10.21037/atm-20-5811 33241003 PMC7576076

[87] Feng J Bao L Wang X Li H Chen Y Xiao W. Low Expression of HIV Genes in Podocytes Accelerates the Progression of Diabetic Kidney Disease in Mice. Kidney Int (2021) 99(4):914–25. doi: 10.1016/j.kint.2020.12.012 PMC8006538 33359498

[88] Zheng XP Nie Q Feng J Fan XY Jin YL Chen G. Kidney-Targeted Baicalin-Lysozyme Conjugate Ameliorates Renal Fibrosis in Rats With Diabetic Nephropathy Induced by Streptozotocin. BMC Nephrol (2020) 21(1):174. doi: 10.1186/s12882-020-01833-6 32398108 PMC7216346

[89] Du YG Wang LP Qian JW Zhang KN Chai KF. Panax Notoginseng Saponins Protect Kidney From Diabetes by Up-Regulating Silent Information Regulator 1 and Activating Antioxidant Proteins in Rats. Chin J Integr Med (2016) 22(12):910–7. doi: 10.1007/s11655-015-2446-1 26712211

[90] Sun HJ Xiong SP Cao X Cao L Zhu MY Wu ZY. Polysulfide-Mediated Sulfhydration of SIRT1 Prevents Diabetic Nephropathy by Suppressing Phosphorylation and Acetylation of P65 NF-kappaB and STAT3. Redox Biol (2021) 38:101813. doi: 10.1016/j.redox.2020.101813 33279869 PMC7718489

[91] Munoz-Felix JM Gonzalez-Nunez M Martinez-Salgado C Lopez-Novoa JM. TGF-Beta/BMP Proteins as Therapeutic Targets in Renal Fibrosis. Where Have We Arrived After 25 Years of Trials and Tribulations? Pharmacol Ther (2015) 156:44–58. doi: 10.1016/j.pharmthera.2015.10.003 26493350

[92] Meng XM Nikolic-Paterson DJ Lan HY. TGF-Beta: The Master Regulator of Fibrosis. Nat Rev Nephrol (2016) 12(6):325–38. doi: 10.1038/nrneph.2016.48 27108839

[93] Sutariya B Jhonsa D Saraf MN. TGF-Beta: The Connecting Link Between Nephropathy and Fibrosis. Immunopharmacol Immunotoxicol (2016) 38(1):39–49. doi: 10.3109/08923973.2015.1127382 26849902

[94] Frangogiannis N. Transforming Growth Factor-Beta in Tissue Fibrosis. J Exp Med (2020) 217(3):e20190103. doi: 10.1084/jem.20190103 32997468 PMC7062524

[95] Xue M Li Y Hu F Jia YJ Zheng ZJ Wang L. High Glucose Up-Regulates microRNA-34a-5p to Aggravate Fibrosis by Targeting SIRT1 in HK-2cells. Biochem Biophys Res Commun (2018) 498(1):38–44. doi: 10.1016/j.bbrc.2017.12.048 29371016

[96] Yu WC Huang RY Chou TC. Oligo-Fucoidan Improves Diabetes-Induced Renal Fibrosis *via* Activation of Sirt-1, GLP-1R, and Nrf2/HO-1: An *In Vitro* and *In Vivo* Study. Nutrients (2020) 12(10):1–15. doi: 10.3390/nu12103068 PMC7650749 33049944

[97] Li C Cai F Yang Y Zhao X Wang C Li J. Tetrahydroxystilbene Glucoside Ameliorates Diabetic Nephropathy in Rats: Involvement of SIRT1 and TGF-Beta1 Pathway. Eur J Pharmacol (2010) 649(1-3):382–9. doi: 10.1016/j.ejphar.2010.09.004 20854812

[98] Zhang J Zhang L Zha D Wu X. Inhibition of Mirna135a5p Ameliorates TGFbeta1induced Human Renal Fibrosis by Targeting SIRT1 in Diabetic Nephropathy. Int J Mol Med (2020) 46(3):1063–73. doi: 10.3892/ijmm.2020.4647 PMC7387088 32705273

[99] Wang X Ji T Li X Qu X Bai S. FOXO3a Protects Against Kidney Injury in Type II Diabetic Nephropathy by Promoting Sirt6 Expression and Inhibiting Smad3 Acetylation. Oxid Med Cell Longev (2021) 2021:5565761. doi: 10.1155/2021/5565761 34122724 PMC8172321

[100] Huang XF Chen JZ. Obesity, the PI3K/Akt Signal Pathway and Colon Cancer. Obes Rev (2009) 10(6):610–6. doi: 10.1111/j.1467-789X.2009.00607.x 19527447

[101] du Rusquec P Blonz C Frenel JS Campone M. Targeting the PI3K/Akt/mTOR Pathway in Estrogen-Receptor Positive HER2 Negative Advanced Breast Cancer. Ther Adv Med Oncol (2020) 12:1758835920940939. doi: 10.1177/1758835920940939 32782489 PMC7388095

[102] Burgering BM Kops GJ. Cell Cycle and Death Control: Long Live Forkheads. Trends Biochem Sci (2002) 27(7):352–60. doi: 10.1016/s0968-0004(02)02113-8 12114024

[103] Hussein MM Mahfouz MK. Effect of Resveratrol and Rosuvastatin on Experimental Diabetic Nephropathy in Rats. BioMed Pharmacother (2016) 82:685–92. doi: 10.1016/j.biopha.2016.06.004 27470412

[104] Wang X Meng L Zhao L Wang Z Liu H Liu G. Resveratrol Ameliorates Hyperglycemia-Induced Renal Tubular Oxidative Stress Damage *via* Modulating the SIRT1/FOXO3a Pathway. Diabetes Res Clin Pract (2017) 126:172–81. doi: 10.1016/j.diabres.2016.12.005 28258028

[105] Wu L Zhang Y Ma X Zhang N Qin G. The Effect of Resveratrol on FoxO1 Expression in Kidneys of Diabetic Nephropathy Rats. Mol Biol Rep (2012) 39(9):9085–93. doi: 10.1007/s11033-012-1780-z 22733486

[106] Yang G Jin L Zheng D Tang X Yang J Fan L. Fucoxanthin Alleviates Oxidative Stress Through Akt/Sirt1/FoxO3alpha Signaling to Inhibit HG-Induced Renal Fibrosis in GMCs. Mar Drugs (2019) 17(12):1–16. doi: 10.3390/md17120702 PMC6950607 31842414

[107] Mori J Patel VB Ramprasath T Alrob OA DesAulniers J Scholey JW. Angiotensin 1-7 Mediates Renoprotection Against Diabetic Nephropathy by Reducing Oxidative Stress, Inflammation, and Lipotoxicity. Am J Physiol Renal Physiol (2014) 306(8):F812–21. doi: 10.1152/ajprenal.00655.2013 24553436

[108] Dusabimana T Kim SR Park EJ Je J Jeong K Yun SP. P2Y2R Contributes to the Development of Diabetic Nephropathy by Inhibiting Autophagy Response. Mol Metab (2020) 42:101089. doi: 10.1016/j.molmet.2020.101089 32987187 PMC7568185

[109] Wang Z Li Y Wang Y Zhao K Chi Y Wang B. Pyrroloquinoline Quinine Protects HK-2cells Against High Glucose-Induced Oxidative Stress and Apoptosis Through Sirt3 and PI3K/Akt/FoxO3a Signaling Pathway. Biochem Biophys Res Commun (2019) 508(2):398–404. doi: 10.1016/j.bbrc.2018.11.140 30502093

[110] Zhou D Zhou M Wang Z Fu Y Jia M Wang X. PGRN Acts as a Novel Regulator of Mitochondrial Homeostasis by Facilitating Mitophagy and Mitochondrial Biogenesis to Prevent Podocyte Injury in Diabetic Nephropathy. Cell Death Dis (2019) 10(7):524. doi: 10.1038/s41419-019-1754-3 31285425 PMC6614416

[111] Kopacz A Kloska D Forman HJ Jozkowicz A Grochot-Przeczek A. Beyond Repression of Nrf2: An Update on Keap1. Free Radic Biol Med (2020) 157:63–74. doi: 10.1016/j.freeradbiomed.2020.03.023 32234331 PMC7732858

[112] Huang K Chen C Hao J Huang J Wang S Liu P. Polydatin Promotes Nrf2-ARE Anti-Oxidative Pathway Through Activating Sirt1 to Resist AGEs-Induced Upregulation of Fibronetin and Transforming Growth Factor-Beta1 in Rat Glomerular Messangial Cells. Mol Cell Endocrinol (2015) 399:178–89. doi: 10.1016/j.mce.2014.08.014 25192797

[113] Zhuang K Jiang X Liu R Ye C Wang Y Wang Y. Formononetin Activates the Nrf2/ARE Signaling Pathway *Via* Sirt1 to Improve Diabetic Renal Fibrosis. Front Pharmacol (2020) 11:616378. doi: 10.3389/fphar.2020.616378 33519483 PMC7845558

[114] Huang K Huang J Xie X Wang S Chen C Shen X. Sirt1 Resists Advanced Glycation End Products-Induced Expressions of Fibronectin and TGF-Beta1 by Activating the Nrf2/ARE Pathway in Glomerular Mesangial Cells. Free Radic Biol Med (2013) 65:528–40. doi: 10.1016/j.freeradbiomed.2013.07.029 23891678

[115] Ma F Wu J Jiang Z Huang W Jia Y Sun W. P53/NRF2 Mediates SIRT1's Protective Effect on Diabetic Nephropathy. Biochim Biophys Acta Mol Cell Res (2019) 1866(8):1272–81. doi: 10.1016/j.bbamcr.2019.04.006 30959066

[116] Huang X Shi Y Chen H Le R Gong X Xu K. Isoliquiritigenin Prevents Hyperglycemia-Induced Renal Injuries by Inhibiting Inflammation and Oxidative Stress *via* SIRT1-Dependent Mechanism. Cell Death Dis (2020) 11(12):1040. doi: 10.1038/s41419-020-03260-9 33288747 PMC7721869

[117] Shao Y Lv C Wu C Zhou Y Wang Q. Mir-217 Promotes Inflammation and Fibrosis in High Glucose Cultured Rat Glomerular Mesangial Cells *via* Sirt1/HIF-1alpha Signaling Pathway. Diabetes Metab Res Rev (2016) 32(6):534–43. doi: 10.1002/dmrr.2788 26891083

[118] Li A Peng R Sun Y Liu H Peng H Zhang Z. LincRNA 1700020I14Rik Alleviates Cell Proliferation and Fibrosis in Diabetic Nephropathy *via* miR-34a-5p/Sirt1/HIF-1alpha Signaling. Cell Death Dis (2018) 9(5):461. doi: 10.1038/s41419-018-0527-8 29700282 PMC5919933

[119] Sun X Huang K Haiming X Lin Z Yang Y Zhang M. Connexin 43 Prevents the Progression of Diabetic Renal Tubulointerstitial Fibrosis by Regulating the SIRT1-HIF-1alpha Signaling Pathway. Clin Sci (Lond) (2020) 134(13):1573–92. doi: 10.1042/CS20200171 32558900

[120] Liu R Zhong Y Li X Chen H Jim B Zhou MM. Role of Transcription Factor Acetylation in Diabetic Kidney Disease. Diabetes (2014) 63(7):2440–53. doi: 10.2337/db13-1810 PMC4066331 24608443

[121] Kundu A Richa S Dey P Kim KS Son JY Kim HR. Protective Effect of EX-527 Against High-Fat Diet-Induced Diabetic Nephropathy in Zucker Rats. Toxicol Appl Pharmacol (2020) 390:114899. doi: 10.1016/j.taap.2020.114899 31981641

[122] Zitman-Gal T Einbinder Y Ohana M Katzav A Kartawy A Benchetrit S. Effect of Liraglutide on the Janus Kinase/Signal Transducer and Transcription Activator (JAK/STAT) Pathway in Diabetic Kidney Disease in Db/Db Mice and in Cultured Endothelial Cells. J Diabetes (2019) 11(8):656–64. doi: 10.1111/1753-0407.12891 30575282

[123] Wang D Li Y Wang N Luo G Wang J Luo C. 1alpha,25-Dihydroxyvitamin D3 Prevents Renal Oxidative Damage *via* the PARP1/SIRT1/NOX4 Pathway in Zucker Diabetic Fatty Rats. Am J Physiol Endocrinol Metab (2020) 318(3):E343–E56. doi: 10.1152/ajpendo.00270.2019 31891537

[124] Li X Cai W Lee K Liu B Deng Y Chen Y. Puerarin Attenuates Diabetic Kidney Injury Through the Suppression of NOX4 Expression in Podocytes. Sci Rep (2017) 7(1):14603. doi: 10.1038/s41598-017-14906-8 29097815 PMC5668268

[125] Scuto M Trovato Salinaro A Modafferi S Polimeni A Pfeffer T Weigand T. Carnosine Activates Cellular Stress Response in Podocytes and Reduces Glycative and Lipoperoxidative Stress. Biomedicines (2020) 8(6):1–14. doi: 10.3390/biomedicines8060177 PMC7344982 32604897

[126] Peters V Calabrese V Forsberg E Volk N Fleming T Baelde H. Protective Actions of Anserine Under Diabetic Conditions. Int J Mol Sci (2018) 19(9):1–12. doi: 10.3390/ijms19092751 PMC6164239 30217069

[127] Zeng S Wu X Chen X Xu H Zhang T Xu Y. Hypermethylated in Cancer 1 (HIC1) Mediates High Glucose Induced ROS Accumulation in Renal Tubular Epithelial Cells by Epigenetically Repressing SIRT1 Transcription. Biochim Biophys Acta Gene Regul Mech (2018) 1861(10):917–27. doi: 10.1016/j.bbagrm.2018.08.002 30496037

[128] Wang XX Wang D Luo Y Myakala K Dobrinskikh E Rosenberg AZ. FXR/TGR5 Dual Agonist Prevents Progression of Nephropathy in Diabetes and Obesity. J Am Soc Nephrol (2018) 29(1):118–37. doi: 10.1681/ASN.2017020222 PMC5748904 29089371

[129] Umino H Hasegawa K Minakuchi H Muraoka H Kawaguchi T Kanda T. High Basolateral Glucose Increases Sodium-Glucose Cotransporter 2 and Reduces Sirtuin-1 in Renal Tubules Through Glucose Transporter-2 Detection. Sci Rep (2018) 8(1):6791. doi: 10.1038/s41598-018-25054-y 29717156 PMC5931531

[130] Tang LX Wang B Wu ZK. Aerobic Exercise Training Alleviates Renal Injury by Interfering With Mitochondrial Function in Type-1 Diabetic Mice. Med Sci Monit (2018) 24:9081–9. doi: 10.12659/MSM.912877 PMC6302662 30551123

[131] Ogura Y Kitada M Xu J Monno I Koya D. CD38 Inhibition by Apigenin Ameliorates Mitochondrial Oxidative Stress Through Restoration of the Intracellular NAD(+)/NADH Ratio and Sirt3 Activity in Renal Tubular Cells in Diabetic Rats. Aging (Albany NY) (2020) 12(12):11325–36. doi: 10.18632/aging.103410 PMC7343471 32507768

[132] Li J Liu H Takagi S Nitta K Kitada M Srivastava SP. Renal Protective Effects of Empagliflozin *via* Inhibition of EMT and Aberrant Glycolysis in Proximal Tubules. JCI Insight (2020) 5(6):1–17. doi: 10.1172/jci.insight.129034 PMC7213787 32134397

[133] Li J Li N Yan S Lu Y Miao X Gu Z. Liraglutide Protects Renal Mesangial Cells Against Hyperglycemiamediated Mitochondrial Apoptosis by Activating the ERKYap Signaling Pathway and Upregulating Sirt3 Expression. Mol Med Rep (2019) 19(4):2849–60. doi: 10.3892/mmr.2019.9946 30816450

[134] Wang XX Edelstein MH Gafter U Qiu L Luo Y Dobrinskikh E. G Protein-Coupled Bile Acid Receptor TGR5 Activation Inhibits Kidney Disease in Obesity and Diabetes. J Am Soc Nephrol (2016) 27(5):1362–78. doi: 10.1681/ASN.2014121271 PMC4849814 26424786

[135] Feng J Lu C Dai Q Sheng J Xu M. SIRT3 Facilitates Amniotic Fluid Stem Cells to Repair Diabetic Nephropathy Through Protecting Mitochondrial Homeostasis by Modulation of Mitophagy. Cell Physiol Biochem (2018) 46(4):1508–24. doi: 10.1159/000489194 29689547

[136] Muraoka H Hasegawa K Sakamaki Y Minakuchi H Kawaguchi T Yasuda I. Role of Nampt-Sirt6 Axis in Renal Proximal Tubules in Extracellular Matrix Deposition in Diabetic Nephropathy. Cell Rep (2019) 27(1):199–212.e5. doi: 10.1016/j.celrep.2019.03.024 30943401

[137] Wang X Lin B Nie L Li P. microRNA-20b Contributes to High Glucose-Induced Podocyte Apoptosis by Targeting SIRT7. Mol Med Rep (2017) 16(4):5667–74. doi: 10.3892/mmr.2017.7224 28849008

[138] Howitz KT Bitterman KJ Cohen HY Lamming DW Lavu S Wood JG. Small Molecule Activators of Sirtuins Extend Saccharomyces Cerevisiae Lifespan. Nature (2003) 425(6954):191–6. doi: 10.1038/nature01960 12939617

[139] Domi E Hoxha M Prendi E Zappacosta B. A Systematic Review on the Role of SIRT1 in Duchenne Muscular Dystrophy. Cells (2021) 10(6):1–28. doi: 10.3390/cells10061380 PMC8229470 34205021

[140] Scher MB Vaquero A Reinberg D. SirT3 is a Nuclear NAD+-Dependent Histone Deacetylase That Translocates to the Mitochondria Upon Cellular Stress. Genes Dev (2007) 21(8):920–8. doi: 10.1101/gad.1527307 PMC1847710 17437997

[141] Akter R Afrose A Rahman MR Chowdhury R Nirzhor SSR Khan RI. A Comprehensive Analysis Into the Therapeutic Application of Natural Products as SIRT6 Modulators in Alzheimer's Disease, Aging, Cancer, Inflammation, and Diabetes. Int J Mol Sci (2021) 22(8):1–23. doi: 10.3390/ijms22084180 PMC8073883 33920726

[142] Gu J Yang M Qi N Mei S Chen J Song S. Olmesartan Prevents Microalbuminuria in db/db Diabetic Mice Through Inhibition of Angiotensin II/p38/SIRT1-Induced Podocyte Apoptosis. Kidney Blood Press Res (2016) 41(6):848–64. doi: 10.1159/000452588 27871084

[143] Kumar GS Kulkarni A Khurana A Kaur J Tikoo K. Selenium Nanoparticles Involve HSP-70 and SIRT1 in Preventing the Progression of Type 1 Diabetic Nephropathy. Chem Biol Interact (2014) 223:125–33. doi: 10.1016/j.cbi.2014.09.017 25301743

[144] Lo CS Shi Y Chenier I Ghosh A Wu CH Cailhier JF. Heterogeneous Nuclear Ribonucleoprotein F Stimulates Sirtuin-1 Gene Expression and Attenuates Nephropathy Progression in Diabetic Mice. Diabetes (2017) 66(7):1964–78. doi: 10.2337/db16-1588 PMC5482081 28424160

[145] Sun Z Ma Y Chen F Wang S Chen B Shi J. miR-133b and miR-199b Knockdown Attenuate TGF-beta1-iIduced Epithelial to Mesenchymal Transition and Renal Fibrosis By Targeting SIRT1 in Diabetic Nephropathy. Eur J Pharmacol (2014) 223:125–33. doi: 10.1016/j.cbi.2014.09.017 30125566

[146] Zhu H Fang Z Chen J Yang Y Gan J Luo L. PARP-1 and SIRT-1 Are Interacted in Diabetic Nephropathy By Activating AMPK/PGC-1alpha Signaling Pathway. Diabetes Metab Syndr Obes (2021) 14:355–66. doi: 10.2147/DMSO.S291314 PMC7846827 33531822

[147] Zhou L Xu DY Sha WG Shen L Lu GY. Long Non-Coding RNA MALAT1 Interacts With Transcription Factor Foxo1 to Regulate SIRT1 Transcription in High Glucose-Induced HK-2 Cells Injury. Biochem Biophys Res Commun (2018) 503(2):849–55. doi: 10.1016/j.bbrc.2018.06.086 29928873

[148] Huang KP Chen C Hao J Huang JY Liu PQ Huang HQ. AGEs-RAGE System Down-Regulates Sirt1 Through the Ubiquitin-Proteasome Pathway to Promote FN and TGF-Beta1 Expression in Male Rat Glomerular Mesangial Cells. Endocrinology (2015) 156(1):268–79. doi: 10.1210/en.2014-1381 25375034

[149] Wen D Huang X Zhang M Zhang L Chen J Gu Y. Resveratrol Attenuates Diabetic Nephropathy via Modulating Angiogenesis. PLoS One (2013) 8(12):1–12. doi: 10.1371/journal.pone.0082336 PMC3849393 24312656

